# Immune responses drive chorioretinitis and retinal pathology after neonatal CMV infection

**DOI:** 10.1126/sciadv.adn6379

**Published:** 2024-11-20

**Authors:** Jessica L. McCord, John Y. S. Han, Ross E. Staudt, Nancy J. Philp, Christopher M. Snyder

**Affiliations:** ^1^ Department of Microbiology and Immunology, Jefferson Center for Vaccines and Pandemic Preparedness, Sidney Kimmel Medical College, Sidney Kimmel Cancer Center, Thomas Jefferson University, Philadelphia, PA, USA.; ^2^ Department of Pathology, Anatomy and Cell Biology, Sidney Kimmel Medical College, Sidney Kimmel Cancer Center, Thomas Jefferson University, Philadelphia, PA, USA.

## Abstract

Human cytomegalovirus (CMV) causes a common congenital infection leading to long-term neurological impairments including brain, cochlear, and ocular pathology. Infection of newborn mice with murine (M)CMV is an established model of neuropathology caused by congenital CMV infection, with recent work suggesting that brain pathology may be driven by immune responses. In the eye, however, CMV retinitis is thought to result from virus-driven necrosis in the absence of T cell responses. We found that MCMV infection of newborn mice recapitulates human eye disease after congenital CMV infection, including focal chorioretinitis, inflamed vasculature, and disrupted blood-retinal barriers. Moreover, infection drove extensive T cell infiltration of the retina and marked gliosis. Blocking immune responses generally, or via targeting the chemokine receptor CXCR3, did not exacerbate retinal disease but instead prevented pathology despite retinal MCMV infection. Thus, our data establish this model for studies of congenital retinal disease and show that the immune system drives pathology in the neonatal eye after MCMV infection.

## Teaser

Immune pathology drives retinal disease in an animal model of congenital cytomegalovirus infection.

## INTRODUCTION

Human cytomegalovirus (HCMV) is a β-herpesvirus that causes a common congenital (in utero) infection, affecting approximately 4 to 6 in every 1000 births, making it the most common congenital infection in the United States (*1*, *2*). Congenital HCMV (cHCMV) infection leads to long-term neurological impairments, with approximately 20 to 30% of children developing hearing loss, vestibular defects, cognitive defects, and/or vision impairment (*2*). These neurological defects can persist throughout life (*3*–*5*). Moreover, since HCMV persists for life after infection, a substantial number of asymptomatic newborns can develop symptoms later in development (*2*, *6*, *7*). Despite the high prevalence of HCMV, little is known about the mechanisms of disease in affected tissues. Currently, the only clinical intervention is antiviral treatment, which is only effective during active viral replication and may not address the course of disease.

HCMV only infects humans and cannot be used in any animal models. However, investigators have modeled cHCMV infection of the brain by intracranial injections of murine (M)CMV into fetal, newborn, or adult mice (*2*, *8*–*21*) or intraperitoneal (ip) infections of newborn mice (<24 hours old) (*22*–*31*). This latter model, originally developed by Jonjic and Britt, has two major advantages for studying congenital CMV disease: (i) Newborn mice are developmentally similar to humans in the late second/early third trimester—a time when HCMV commonly infects the fetus—and (ii) MCMV reaches neural tissues from the circulation, as would be expected from a placental transmission. Critically, these and other investigators have shown that MCMV infection of newborn mice recapitulates parts of natural human infection with evidence of hearing loss and cognitive/developmental deficits (*32*). Moreover, some data have suggested that disease in the brain may be driven by inflammation, possibly interferon-γ (IFN-γ) and/or tumor necrosis factor–α, and involves influx of innate immune cells and activation of microglia (*23*–*27*).

While this newborn infection model has been used to study brain infections, it is unknown whether there is any involvement of the eye. Approximately 20% of symptomatic children develop vision impairment after cHCMV infection (*3*–*6*, *33*). Typically, this is characterized by chorioretinitis with retinal lesions that occur randomly throughout the retina and can lead to permanent retinal scarring and optic neuritis leading to optic nerve atrophy and cortical visual impairment (*4*, *33*, *34*). In addition, inflammation in the retinal blood vessels has been described, manifesting as “frosted branch angiitis” (*34*–*36*). However, the mechanisms behind these pathologies are unclear. The retina is a neural tissue, derived from the neural tube, separated from the blood supply by the blood-retinal barriers (BRBs), and considered to be part of the central nervous system (CNS) (*37*–*40*). However, while there are similarities between the brain and eye, there are also many fundamental differences, including the existence of two distinct BRBs that form during the period of infection (*41*) and later than the blood-brain barrier (*42*), the presence of Müller glial cells, which are known to respond to infections (*43*, *44*), the constant cellular damage caused by light, and multiple immune modulating mechanisms (*45*, *46*). Unlike the immune pathology described for the brain, previous work on CMV-driven retinitis in adults has suggested that T cells are protective of the eye and that CMV induces retinal necrosis in the absence of an adequate T cell response (*47*–*56*). Thus, it is unclear how viral infection progresses or is controlled in the presence of an immature but functional fetal or neonatal immune response and how damage occurs during development of this critical CNS tissue.

We used the MCMV model of congenital infection originally described by Jonjic and Britt and show here that MCMV drives a focal chorioretinitis with marked immune infiltration, glial cell activation, and pathology in the retina that closely mirrors what is observed in humans. Critically, our data show that the immune response drives pathology and can be targeted to prevent most disease manifestations. This suggests that therapies targeting immune function may be essential to reduce disease burden after congenital CMV infection.

## RESULTS

### Neonatal infection with MCMV leads to eye infection and retinal pathology

**Fig. 1.**
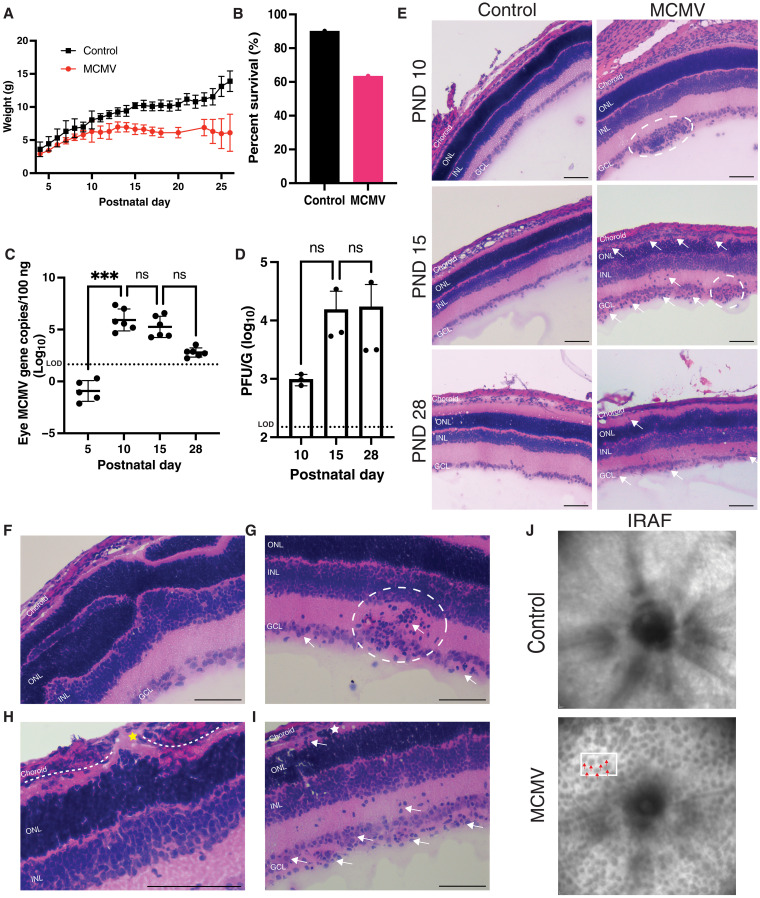
MCMV infection disseminates to the eye and infection induces retinal pathologies. (**A**) Weight gain measured in grams (g) over time of mouse neonates infected with MCMV (*n* = 3 to 15) or controls (*n* = 3 to 21). (**B**) Representative (*n* ≥ 24) survival at PND15 of MCMV infected versus control pups based on the number injected on PND0. (**C**) MCMV genome copies per 100 ng of DNA in ocular tissue over time (*n* = 6 eyes from 6 individual mice per time point). Limit of detection (LOD), 44 genomes/100 ng. (**D**) MCMV titer in ocular tissue over time measured in plaque-forming unit per gram of tissue (*n* = 3 eyes from 3 individual mice per time point). LOD, 150 plaques/g. (**E**) H&E staining of eyes from mice injected with MCMV or PBS controls at PND10, PND15, and PND28 (representative of *n* = 9 to 16 per group). Shown are cellular clustering in the GCL (white dotted circles) and cellular infiltration (white arrows). (**F** to **I**) Specific pathologies of MCMV-infected eyes. (F) Rosette structures of the ONL and inner nuclear layer (INL) at PND10. (G) Cellular accumulation in GCL (white dotted circle) and cellular infiltration (white arrows) at PND15. (H) Breaks in RPE layer (yellow star). White dotted line represents RPE layer. (I). Cellular deposits in photoreceptor layer (white star) and cellular infiltration (white arrows) at PND15. (**J**) cSLO infrared reflectance (IRAF) images of MCMV and control eyes at PND28 (representative of *n* = 3 per group). White box highlighting representative area of photoreceptor infoldings (red arrows). Scale bars, 100 μm. Error bars represent SD. Analyzed by one-way analysis of variance (ANOVA) with multiple comparisons; ***P < 0.001. ns, not significant.

Newborn BALB/c mice were infected with 200 plaque-forming units (PFU) of MCMV strain K181 or an equal volume of phosphate-buffered saline (PBS) for controls, following the protocol developed by Jonjic and Britt to model cMCMV (*31*, *32*, *57*). Infected pups had a reduction in weight gain compared to controls (Fig. 1A) and a decrease in survival (Fig. 1B), as previously reported with this model (*24*). The pups had delayed eye opening compared to controls, with most of the infected eyes remaining closed at postnatal day 15 (PND15), while all control eyes were open by PND12 (table S1). Plaque assays measuring viral titers in the lungs demonstrated a robust systemic infection and confirmed the overall kinetics of viral growth (fig. S1A), similar to previous studies (*24*, *31*). Viral DNA in the eye was below our limit of detection on PND5. By PND10, however, all eyes had substantial levels of virus, which were sustained on PND15 and then reduced by PND28 (Fig. 1C). Plaque assays of the whole eye showed active virus replication through the PND28 time point (Fig. 1D). Thus, MCMV robustly infected ocular tissue in this model.

To determine whether MCMV-infected eyes had any virally induced pathology, eyes from each time point were analyzed histologically by hematoxylin and eosin (H&E) staining (Fig. 1E and fig. S1B). Profound pathology was observed as early as PND10, was more widespread by PND15, and was only moderately resolved by PND28. Similar to what has been described in humans with congenital retinitis (*4*, *33*–*35*), the affected areas appeared to be in random focal regions and were not evident in all sections from a given eye, requiring examination of multiple serial sections (fig. S1C; see Materials and Methods). Nevertheless, every animal had evident pathology (table S1), and both eyes were affected in each animal, albeit with varying degrees of severity. The most prominent pathology observed at PND10 was the accumulation of cells in the ganglion cell layer (GCL; Fig. 1E, white circle) and rosette-like structures in the outer nuclear layer (ONL) disrupting normal lamination (Fig. 1F). By PND15, there were increased frequencies of cellular clusters in the GCL (Fig. 1, E and G, white circle), breaks in the retinal pigment epithelium (RPE) (Fig. 1H, yellow star), cellular infiltration in the inner retina (Fig. 1I, white arrows), and delamination and thinning of retinal layers with photoreceptor layer disruption (Fig. 1E). Although cellular infiltration had decreased by PND28, indicating a resolution phase of the infection, there was still noted retinal thinning, delamination, cell clusters in GCL, and some remaining cellular infiltration (Fig. 1E). Scanning laser ophthalmoscopy (SLO) of live animals at PND28 revealed infoldings in the photoreceptor layer determined by areas of less penetrating light (Fig. 1J, red arrows), which was confirmed by optical coherence tomography (OCT) of the affected areas showing disruption of layer lamination at these foci (fig. S1D).

**Fig. 2.**
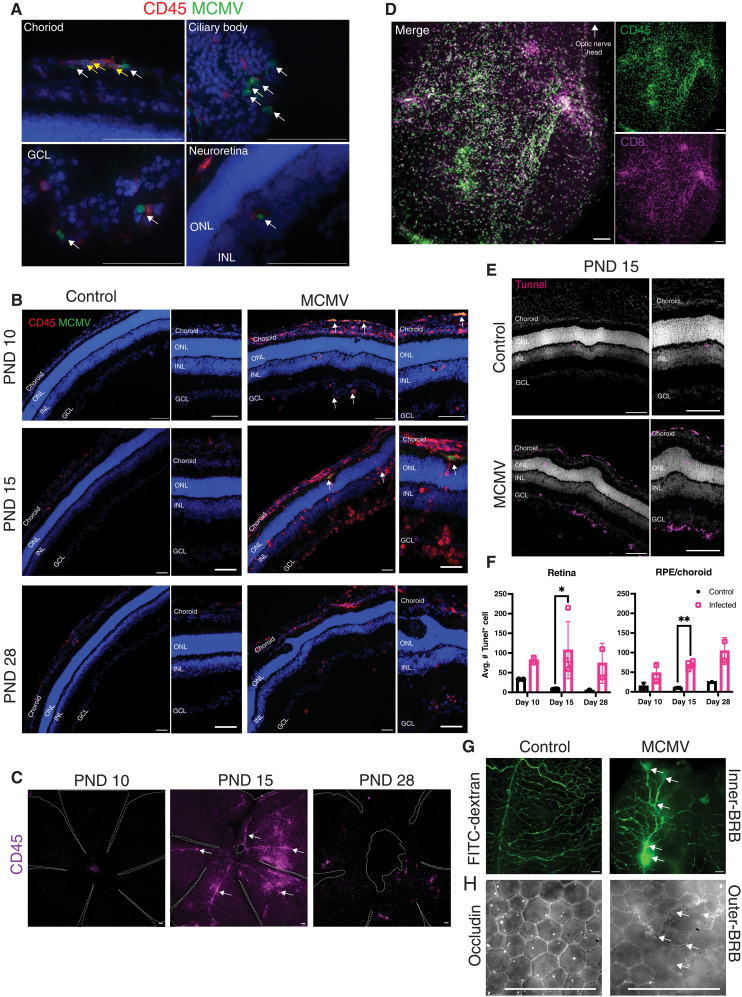
Immune infiltration and cell death in the eyes of MCMV-infected neonates. (**A**) Representative images showing areas of virus-infected cells. MCMV-infected eyes were stained for CD45 and MCMV-IE1 (pp89). White arrows denote MCMV-positive cells, and yellow arrows denote CD45/MCMV double-positive cells. Representative of *n* ≥ 5 mice. (**B**) Immune cell infiltration over time. MCMV and control mice at PND10, PND15, and PND28 stained for CD45 and MCMV-IE1. MCMV-positive cells are indicated by the white arrows. Images on the right-hand side of the panels show higher magnification of selected regions. Representative of *n* ≥ 5. (**C**) Retinal flat mounts showing distribution of CD45^+^ cell infiltration at the indicated time points. Tissue edges were outlined with white dotted lines. White arrows denote retinal vasculature. Representative of *n* = 3 to 5. (**D**) Retinal flat mount stained for CD45^+^ and CD8^+^ cells to identify overlap. Representative of *n* = 3. (**E**) Representative TUNEL staining at PND15 of MCMV-infected and control mice. Images on the right-hand side of the panels show higher magnification of selected regions. Representative of *n* = 5 per group. (**F**) TUNEL^+^ cells were calculated from nine images per mouse in *n* = 3 to 4 infected mice or *n* = 3 control mice. Error bars represent SD. *P < 0.05 **P < 0.01. (**G**) Retinal flat mounts of MCMV and control mice injected with 10,000 Da FITC-dextran. Vasculature breaks are indicated by the white arrows (*n* = 3 mice per group). (**H**) RPE flat mounts of MCMV and control mice stained with occludin to identify RPE tight junctions. Representative images taken from mid-retina. Breaks in occludin are indicated with white arrows (*n* = 4 mice per group). Scale bars, 100 μm.

Next, the regions of the eye with virally infected cells were identified by staining for viral pp89 (encoded by the MCMV IE1 gene). Infected cells were found in all areas of the eye, including the choroid and ciliary body, the RPE, as well as throughout the neuroretina, including the ONL and inner nuclear layer (INLs), inner plexiform layer, and, importantly, the GCL (Fig. 2A and table S2). This is consistent with prior work describing the cells infected by MCMV in the eye (*58*, *59*). Virus-infected cells were evident at both PND10 and PND15 (Fig. 2B), and we frequently observed infected CD45^+^ immune cells in the choroid and ciliary body at these times (Fig. 2B). In addition, we occasionally found pp89 staining in the nucleus of CD45^+^ cells that had infiltrated the neuroretina. Thus, in addition to infecting cells within the eye, MCMV infected some of the infiltrating immune cells either before or after their migration into the retina (fig. S2A). By PND28, no virally infected cells were observed by thin-section histology (Fig. 2B), suggesting ongoing resolution of the infection. Together, these data demonstrate that the cMCMV model enabled infection of the eye that induced severe but focal retinal pathology, including disrupted retinal lamination, infoldings in the photoreceptor layer, and cellular infiltrations.

### MCMV infection drives focal immune infiltration

The pathology described above is consistent with focal chorioretinitis, which has been described in humans with cHCMV infection (*4*, *33*–*35*). In agreement with the H&E staining (Fig. 1E), infiltration of CD45^+^ immune cells began in the outer retinal structures (choroid, iris, and ciliary body) on PND10 but were not present in the neuroretina until PND15 (Fig. 2, B and C). Notably, an accumulation of CD45^+^ cells could be identified in the GCL on PND15, suggesting that the cell clustering observed by H&E (Fig. 1, E, G, and I) included CD45^+^ cells. By PND28, far fewer CD45^+^ cells remained in the neuroretina, again suggesting resolution of the immune response, but residual clusters of cells were still evident in the GCL (Fig. 2B). Whole retinal flat mounts showed CD45^+^ cellular infiltration only in portions of the retina with evident involvement of the retinal vasculature (Fig. 2C, white arrows indicate CD45^+^ cells in the vasculature), confirming the focal nature of these infiltrates. The majority of these infiltrating CD45^+^ cells, ~85%, were CD8^+^ T cells (Fig. 2D and fig. S2, B and C). Clinical observations from human patients described a similar random distribution of the affected areas in the retina and inflammation that follows the retinal vasculature resulting in frosted branch angiitis (*34*–*36*). Moreover, optic neuritis is a frequent complication of congenital CMV infection in humans, and we routinely observed CD8^+^ T cell infiltration of the optic nerve in the cMCMV model (fig. S2E), indicating optic neuritis. Thus, MCMV drove a focal chorioretinitis with involvement of the retinal vasculature and T cell infiltration of the retina and optic nerve.

### Neonatal MCMV infection causes retinal cell death and damage to the BRBs

Next, we used the terminal deoxynucleotidyl transferase–mediated deoxyuridine triphosphate nick end labeling (TUNEL) assay to determine whether infection and immune infiltration were associated with increased cell death. As expected, TUNEL^+^ cells were found in control eyes as cell death is a critical component of retinal development (*60*, *61*). However, a marked increase in TUNEL^+^ cells was evident at PND10, PND15, and PND28. Staining was most evident in the ONL and INL on PND10 but shifted to the choroid and GCL on PND15 and PND28 after the arrival of immune cells (Fig. 2, E and F, and fig. S2D).

Since there was an increased number of immune cells in the inner retina of infected mice on PND15, we hypothesized that the BRBs were compromised. The eye is considered an immune-privileged site where the BRBs limit access to protect the homeostasis of the tissue (*37*–*39*). In a healthy developing neonate, the BRBs are fully developed at approximately PND10 and closed by the time the neonates open their eyes on approximately PND12 (*41*). To evaluate the BRB integrity on PND15, the mice were injected with fluorescein isothiocyanate (FITC)–dextran, and whole retina flat mounts were imaged to visualize any leakage of FITC-dextran into the retina. While the FITC-dextran was confined to retinal vasculature in control mice, there were individual points of FITC-dextran leakage in the eyes of all infected mice, indicating that the BRB was disrupted at these foci (Fig. 2G, white arrows, and table S1). We also directly assessed the integrity of the outer BRB by staining flat mounts of the RPE for occludin, a component of the tight junctions between RPE cells. Breaks in occludin staining were clearly evident in individual foci in every infected mouse (Fig. 2H, white arrows). These data confirm that infection with MCMV disrupted the integrity of both inner and outer BRBs. Collectively, these data show that the cMCMV model recapitulates key features of human disease, with chorioretinitis and optic neuritis, focal disruptions of retinal lamination, focal immune infiltrations that trace the retinal vasculature and are associated with disrupted regions of the BRBs, and marked cell death in the retina, particularly in the GCL.

### MCMV infection induced a gene signature consistent with immune response and dysregulation of retinal homeostasis

To further characterize the response to MCMV infection in a developing eye, we performed bulk RNA sequencing (RNA-seq) of infected and control eyes over time. These data identified approximately 2000 differentially expressed genes (DEGs) across all time points. Unsupervised clustering showed that MCMV-infected groups clustered together at PND 10, 15, and 28, while eyes from infected mice at PND5 were most similar to PND5 controls (fig. S3A). The kinetics of the RNA-seq results clearly recapitulated the course of infection visualized by histological approaches. Counts of DEGs between infected and control mice increased from 143 on PND5 to 617 on PND10, with a peak of 1611 on PND15 and resolved to only 141 DEGs at PND28 (fig. S3B). There were very few significantly down-regulated genes in infected eyes at any of the tested time points.

**Fig. 3.**
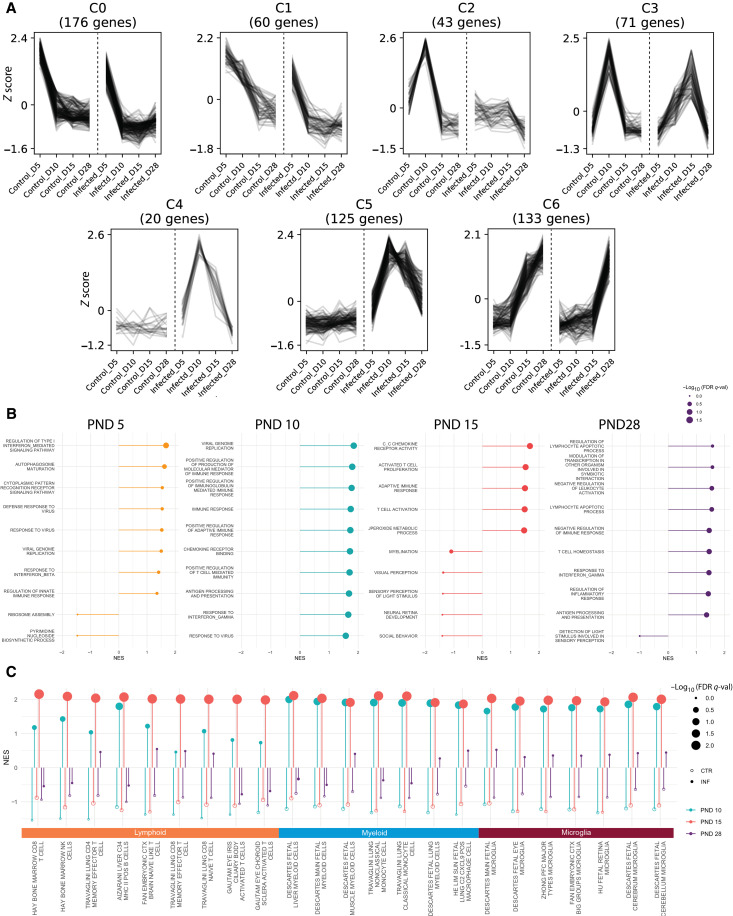
Transcriptional profiling of dysregulated retinal homeostasis and immune activation caused by MCMV. (**A**) Unsupervised coexpression clustering analysis was conducted on differentially expressed gene sets from MCMV-infected and control eyes at all time points. Line graphs of each gene from each cluster over time were plotted with respect to *z* score. Dotted lines separate control from infected conditions in each cluster. (**B**) Differential expression analysis was conducted between MCMV-infected and control eyes at different time points. GSEA was used to generate enrichment for Molecular Signatures Database (MSigDB) C5 GO gene set at each time point. Some of the highest ranked, nonredundant enrichment was selected for each time point and plotted as lollipops by their normalized enrichment score (NES, denoted as line length) and significance [−log10 (FDR *q* val), denoted as dot size]. (**C**) DEGs from eyes at PND10, PND15, and PND28 were analyzed by GSEA to identify specific cellular identity signatures. Each time point and condition was compared to all differential expression data from all of the time points to identify unique cell signatures at a given time (MSibDB C8: Cell type signature gene set). Top enrichments for each cellular category for each time point were plotted as lollipops with NES denoted by line length and [−log10 (FDR *q* val)] denoted as dot size. Open dots indicate control time points and closed dots are MCMV-infected time points. *n* = 3 samples per group with each sample pooled from two mice.

To identify groups of genes with similar kinetics over time, we performed coexpression clustering of the DEGs. This analysis identified six unique clusters that allowed visualization of gene expression changes over time caused by MCMV infection (Fig. 3A and data file S1). Gene ontology (GO) enrichment analyses were then used to identify the biological processes represented by these clusters (fig. S3C). Together, these analyses revealed that MCMV accelerated the down-regulation of pathways involved in tissue development (cluster C1); impaired or delayed the expression of metabolic pathways (clusters C2 and C3); induced the abnormal expression of immune response pathways (clusters C4 and C5); and delayed the expression of normal immune response, immune regulation, stress response, and cell adhesion pathways (cluster C6). Thus, MCMV disrupted normal eye developmental and metabolic processes while markedly inducing immune responses not normally observed in the eye. Next, we compared each time point individually using gene set enrichment analysis (GSEA) against GO gene sets to identify specific biological differences at each stage of MCMV infection. At PND5, pathways involved with viral replication and the type 1 IFN response were positively enriched, while pathways involved in protein synthesis were negatively enriched (Fig. 3B). Thus, despite MCMV loads in the eye that were below our level of detection (Fig. 1C) and no evidence of pathology (fig. S1B), the tissue is already initiating an innate immune response at PND5. By PND10, there was significant positive enrichment for pathways involved in innate immune responses and viral genome replication (Fig. 3B), further corroborating the histology and viral titer data. On PND15, pathways involved with the adaptive immune response were strongly activated (Fig. 3B). While some of these adaptive immune response pathways remained positively enriched at PND28 (control eyes had essentially no expression of adaptive immune genes), we also observed a positive enrichment in processes involved with lymphocyte apoptosis (Fig. 3B), coinciding with greatly reduced immune infiltration detected by histology (Fig. 2, B and C). Extending these analyses to include the Hallmark GO and KEGG pathways, which use larger gene sets and may therefore reveal additional features, confirmed the overall trends (fig. S4, A, B, E, and F) and identified enrichment of cell death pathways at PND10 and PND15 and enrichment of cellular replication pathways at PND15, suggesting a transient increase cell proliferation that aligns with the peak of immune infiltration (fig. S4, D and H).

Although there was a very limited number of down-regulated genes at any of the tested time points, there were negative trends in pathways associated with visual perception beginning at PND15 (Fig. 3B). Although the negative enrichment of these pathways was not statistically significant, it should be noted that these analyses included the whole eye and not isolated retinas. Collectively, these data show that MCMV infection markedly disrupted the normal patterns of gene expression involved with retinal development and metabolism. Moreover, infection drove profound innate and adaptive immune responses that were associated with cell death and were reduced but not resolved even by PND28.

### MCMV infection induces reactive glial cells

Next, we used GSEA and gene set variance analysis (GSVA) to identify differences in the enrichment of pathways for specific cell signature gene sets (Fig. 3C), with a focus on T cells, myeloid cells, and microglia. Microglia are the tissue-resident macrophages in the CNS and are important for retinal homeostasis as well as the first to respond to infection (*62*–*66*). GSEA analyses comparing each time point to all others enabled us to identify the relative kinetics of enrichment across the time points. These data clearly indicated that myeloid and microglia signatures were enriched by PND10 while peak enrichment of T cell signatures occurred at PND15 (Fig. 3C), in agreement with our histological data (Fig. 2). GSVA analyses showed that microglia gene sets were the most significantly variant across the time course, with most microglia pathways showing statistically significant differences in the enrichment between all of the time points (fig. S4I and table S3). In contrast, significant differences in myeloid cell gene sets were most evident between PND28 and the other time points, while T cell gene sets showed the most significant differences between PND15 and PND28 (table S3). These data suggest that infection induced significant microglial activation across the time course.

**Fig. 4.**
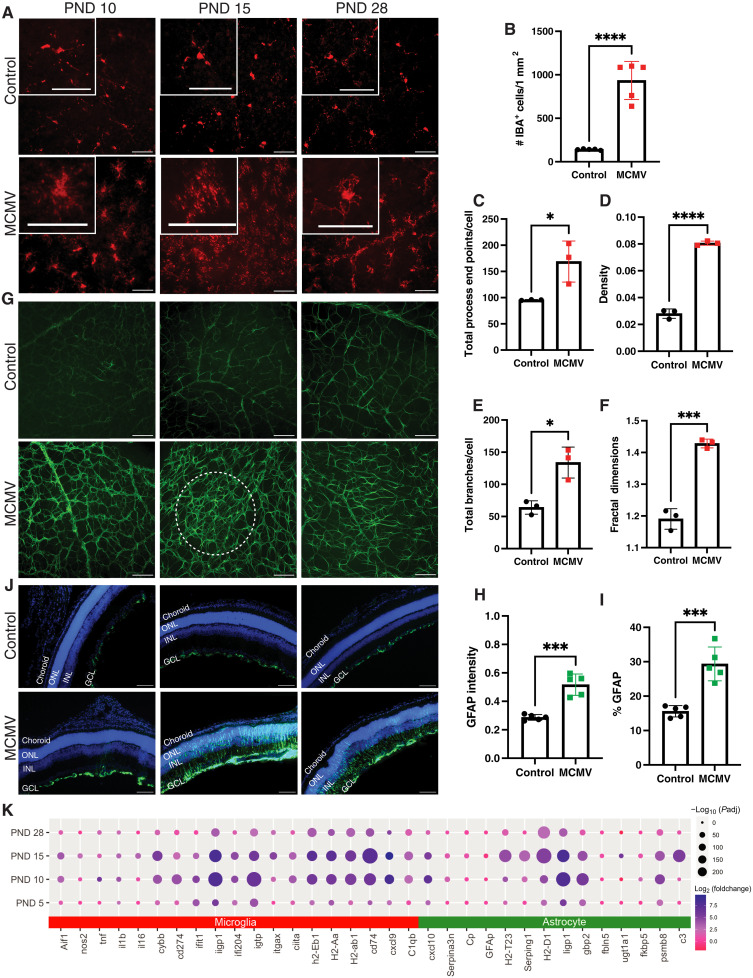
Glial cell activation in response to MCMV infection in the eye. (**A**) Microglia morphology over time. MCMV-infected and control retinal flat mounts stained with Iba1 (representative of *n* = 5 per time point). Scale bars, 100 μm (main image) and 50 μm (insets). (**B**) Average Iba1^+^ cells per 1-mm^2^ area of 4′,6-diamidino-2-phenylindole (DAPI). Counts were averaged from five retinal flat mount images per mouse from *n* = 5 mice. (**C** to **F**) Morphological analysis of Iba1^+^ cells from MCMV and control retinas as assessed by (C) total process end points per cell, (D) total density of each cell, (E) total number of branches per cell, and (F) cell complexity measured by fractal dimensions per cell. Each point shows an averaged value derived from *n* > 15 cells from an individual mouse and was graphed from *n* = 3 to 5 individual mice per analysis. Significance was analyzed by unpaired *t* test; *P < 0.05 ***P < 0.001 ****P < 0.0001. (**G**) Astrocyte distribution over time as assessed on retinal flat mounts stained for GFAP. Dotted white circle denotes an area of increased staining density. (**H** and **I**) Activation of astrocytes as assessed by (H) intensity of GFAP staining in MCMV-infected and control retinas and (I) percent coverage of GFAP cells per DAPI area of MCMV-infected and control retinas. Data show an average derived from five images per animal and *n* = 5 mice per group. Asterisks indicate statistical significance as in (C) to (F). (**J**) Müller glial cell activation. Shown are sections from MCMV-infected and control samples stained with GFAP (representative of *n* = 3 to 5 mice per group). (**K**) Differential expression analysis of genes associated with microglia and astrocyte activation. Genes indicating microglia and astrocyte activation were plotted. Color indicates log_2_ fold change in infected mice compared to controls at the same time point. Dot size indicates significance [log_10_(padj)].

Glial cells in the retina, including microglia, astrocytes, and Müller glia, are important for development, signal transduction, tissue homeostasis, and regulating immune responses (*43*, *44*, *62*, *63*, *65*, *67*). Activated microglia adopt a more compact shape, with shorter processes (*68*, *69*), and, indeed, microglia appeared more compact in MCMV-infected retinas compared to controls at each of the time points (Fig. 4A). Quantification of Iba1^+^ microglia numbers and morphology at PND15 revealed significant increases in the numbers of microglia and their activation state as assessed by the numbers of processes, the cell density, the number of branches per cell, and the complexity of the cells (Fig. 4, B to F, and fig. S5, A and B). Activated microglia also move to sites of damage in the eye (*70*–*72*). Morphologically activated microglia were evident in the subretinal space in the eyes of infected mice but not in controls (fig. S5, C and D), indicating a response to photoreceptor or RPE damage. This accumulation of activated microglia in the subretinal space was also observed on PND28 by visualizing hyperfluorescent foci by SLO imaging (fig. S5E) (*73*). We sometimes observed an infected cell in the vicinity of these activated microglia (fig. S5F). Moreover, microglia with pp89-positive puncta in their cytoplasm could be found in this subretinal area, indicating engulfment of debris from infected cells (fig. S5G). Notably, we did not observe infection of the microglia themselves (i.e., pp89-stained nuclei of microglia). Together, these data suggest that microglia are moving to areas with infected cells in the eye and engulfing debris from these cells.

Other glial cells in the eye are also known to respond to damage or infection, as well as contribute to neurotoxic pathologies (*74*–*79*). The glial fibrillary acidic protein (GFAP) is expressed by astrocytes, and expression is strongly increased on reactive astrocytes, while Müller glial cells lack GFAP until activation. MCMV infection induced the strong up-regulation of GFAP on astrocytes in the GCL and disrupted their normal patterning across all time points (Fig. 4, G to I), suggesting that reactive astrocytes were induced early in the infection and sustained throughout the time course. GFAP expression was also induced on Müller glial cells, which traverse the retinal layers, with a significant increase in expression evident at PND15, consistent with the peak of cellular infiltration (Fig. 4J). In addition, we found significant differential expression of genes involved in microglia and astrocyte activation in MCMV-infected mice (Fig. 4K), most of which were up-regulated at PND10 and PND15. Notably, some of these genes have been linked with neurotoxic glia, including major histocompatibility complex II (MHC-II) (H2-A/E genes and CD74 invariant chain), Aif1 (encodes Iba1), nos2 [encodes inducible nitric oxide synthase (iNos)] and C3, or with IFN-γ stimulation, including iigp1, igtp, gbp2, serping1, CXCL9, and CXCL10 (*69*, *77*, *79*–*81*). Overall, these data show that retinal glial cells are activated in response to MCMV infection in the eye and may be skewed toward a pathologic activation state.

### MCMV infection induces the formation of glial nodules in the GCL, which attract CD8^+^ T cells and express a neurotoxic phenotype

**Fig. 5.**
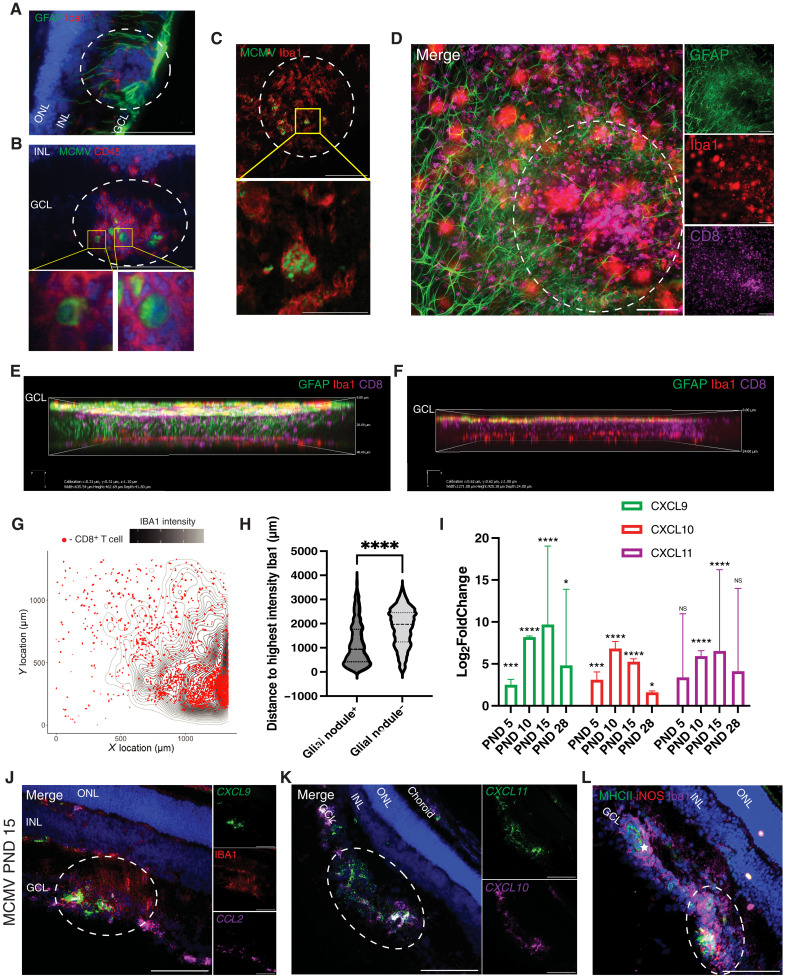
Glial nodules form in response to MCMV infection in the eye and recruit CD8^+^ T cells. (**A**) Nodules in the GCL of MCMV-infected eyes are composed of Iba1^+^ microglia surrounded by GFAP^+^ astrocytes and Müller glial cells. (**B**) Representative image of MCMV^+^ cells in the center of the nodule in GCL. Below, zooms of MCMV^+^ cell with cytosolic staining (left) and nuclear staining (right). (**C**) Retinal flat mount of glial nodule stained for MCMV (pp89) and Iba1^+^ microglia and zoom in of designated region below. (**D**) Retinal flat mount of glial nodule stained for GFAP^+^ astrocytes, Iba1^+^ microglia, and CD8^+^ T cells. (**E** and **F**) Three-dimensional (3D) projection of retinal flat mounts from an MCMV-infected eye stained with GFAP, Iba1, and CD8. Showing (E) an area with a glial nodule and (F) and area with no nodule formation. (**G**) Representative contour graph of a retinal flat mount showing the distribution of Iba1^+^ staining intensity overlayed with the spatial distribution of CD8^+^ cells. (**H**) Violin plot of the distance of each CD8^+^ T cell to highest intensity Iba1^+^ point as analyzed in (G). Analyzed by unpaired *t* test. (**I**) Differential gene expression of the indicated chemokines overtime from the RNA-seq analyses described in Fig. 4. Analyzed by one-way ANOVA with multiple comparisons. (**J**) In situ hybridization (RNAScope) for expression of *CXCL9* and *CCL2* mRNA in glial nodules and costained with Iba1. (**K**) In situ hybridization for expression of *CXCL10* and *CXCL11*. (**L**) Representative image from an MCMV-infected eye stained with MHCII, iNOS, and Iba1. White star indicates a blood vessel. Scale bars, 100 μm (throughout) and 100 and 25 μm (C), respectively. Dotted white circles indicate glial nodule. All Images are representative of *n* = 3 to 6 mice. *P* < 0.05 ***P < 0.001 ****P < 0.0001.

Nodules of activated glial cells were previously observed in studies of MCMV or guinea pig CMV infection in the brain, where focal glial nodules were observed around areas of infected cells (*11*, *28*, *82*–*86*). Similar nodules have been described in humans with CMV encephalitis (*87*). In the eyes of MCMV-infected mice, nodules of nuclei were observed focally in the GCL (Fig. 1, E and G) and frequently contained CD45^+^ immune cells (Fig. 2B). These nodules in the retina were composed of Iba1^+^ CD45^dim^ microglia and surrounded by GFAP^+^ astrocytes and Müller glial cells (Fig. 5A and fig. S6A). pp89^+^ virally infected cells were sometimes observed in the center of these nodules. In some cases, staining localized in the nucleus, indicating infected cells within the nodule (Fig. 5B). However, using confocal microscopy, we also observed Iba1^+^ microglia containing viral antigen in phagosomal-like inclusions, indicating a role for these cells in clearing infected debris within nodules (Fig. 5C). These nodules were clearly focal and randomly distributed when visualized by whole retinal flat mounts (fig. S6B) and ranged in size with the most abundant and largest nodules observed at PND15 (fig. S6, C and D). Astrocytes were cleared from the nodule center and accumulated around the edges where GFAP^+^ Müller glial processes were also evident (Fig. 5, D and E, and movie S1). Confocal imaging of these nodules showed that accumulation of cells was mostly confined to the GCL but that the Müller glial processes around the nodule extended through all layers of the retina, while areas with no nodules tended to lack activated GFAP^+^ Müller glia but still had microglia in the subretinal space (Fig. 5F). Microglia and CD8^+^ T cells were most abundant in the center of the nodules (Fig. 5, D and E, and movie S1) and CD8^+^ T cells appeared to be particularly enriched within the nodules compared to areas with no nodule formation (Fig. 5D). To quantify this, we measured the distance of each T cell to the location of the nodule as defined by the highest intensity of Iba1 staining in the image. These analyses demonstrated that CD8^+^ T cells accumulated significantly closer to microglia where glial nodules formed, compared to areas of the retina with no nodules (Fig. 5, G and H, and fig. S6E).

The lymphocyte chemoattractants CXCL9, CXCL10, and CXCL11 are major drivers of T cell migration and were significantly up-regulated in the eye as assessed by RNA-seq (Fig. 5I). RNA scope in situ hybridization showed that *CXCL9*, *CXCL10*, and *CXCL11* were highly expressed in the choroid and the inner retina of infected mice (Fig. 5, J and K) but were absent from control uninfected animals (fig. S7, A and B). In the neuroretina on PND15 after infection, expression of these chemokines was almost exclusively localized within glial nodules in the GCL (Fig. 5, J and K, white dotted circle). In contrast, CCL2, which is known to be expressed by activated astrocytes (*88*–*90*), was expressed along the GCL where astrocytes reside and not exclusively in the nodules (Fig. 5J). Expression of CXCL9 is a marker of activated and potentially neurotoxic microglia (*91*, *92*), and *CXCL9* transcript expression colocalized with Iba1 staining in the GCL (fig. S7C). Likewise, MHC-II and iNOS, which are also associated with neurotoxic microglia, were both observed on Iba1^+^ cells in glial nodules (Fig. 5L). These molecules are known to be induced by IFN-γ, and most of the *IFN*-γ expression was localized to glial nodules of MCMV-infected eyes (fig. S7, D to F). Overall, these data suggest that glial nodules were forming around MCMV-infected cells, leading to the recruitment of CD8^+^ T cells into the neuroretina, with microglia in the nodules engulfing infected cell debris and expressing IFN-γ–stimulated genes.

### Blocking CXCR3 prevents immune infiltration and retinal pathology

**Fig. 6.**
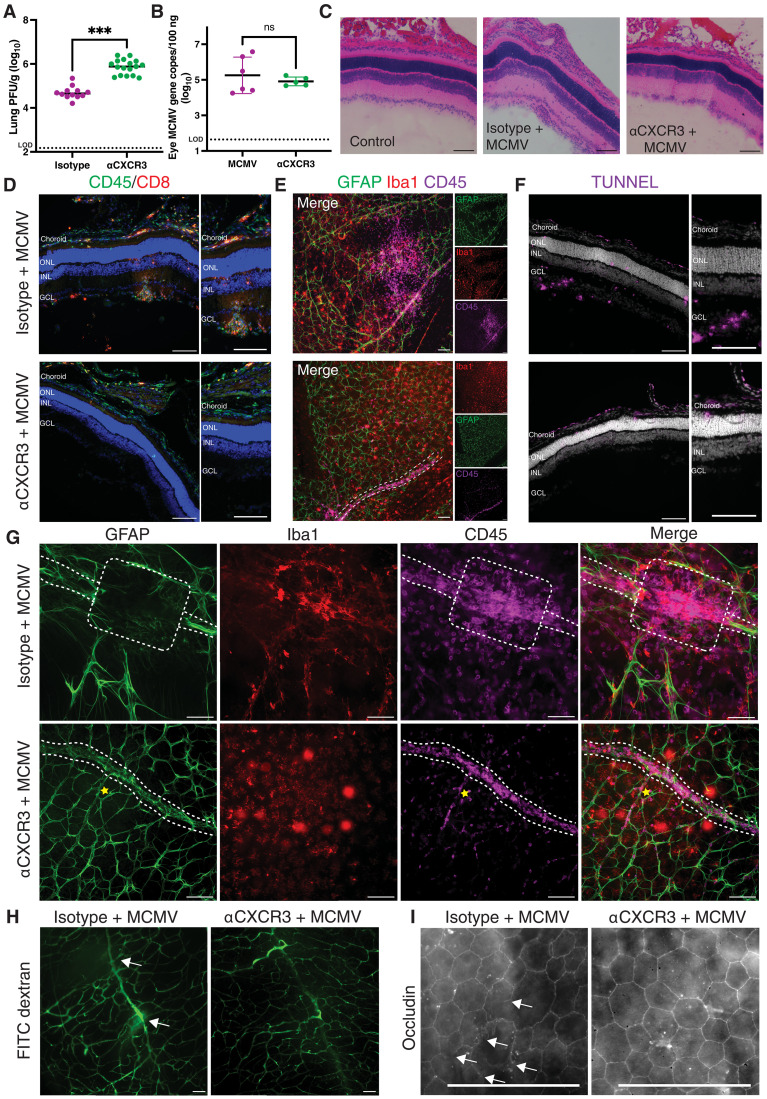
Blocking CXCR3 prevents retinal pathology and immune infiltration while maintaining BRB integrity. (**A**) MCMV titer measured by plaque assay from lung tissue on PND15 from mice infected with MCMV and either treated with αCXCR3 or isotype control antibody. Data are pooled from *n* = 12 to 17 mice per group. LOD, 150 plaques/g. Analyzed by unpaired *t* test; ***P < 0.001 . (**B**) MCMV genome copies per 100 ng of DNA in ocular tissue of mice treated with or without αCXCR3 [*n* = 5 to 6 mice per group, analyzed as in (A)]. LOD, 44 genomes/g. (**C**) H&E-stained sections from control (uninfected) mice or MCMV-infected mice treated with isotype control or and αCXCR3 antibodies. Images are representative of *n* = 8 mice per group. (**D** to **I**) Eyes from MCMV-infected mice treated with isotype-control or αCXCR3 antibodies (*n* = 3 to 8 mice per group) showing: (D) CD45^+^ and CD8^+^ cells on thin sections. Images on the right-hand side show an enlarged region). (E) GFAP, Iba1, and CD45 staining on retinal flat mounts. The white dotted line outlines the retinal vasculature in the bottom image. (F) TUNEL staining of thin sections. Images on the right-hand side show an enlarged region. (G) Higher magnification images of GFAP, Iba1, and CD45 staining on retinal flat mounts showing the presence of CD45^+^ cells in the vasculature. White line outline denotes vasculature. White box in top panels indicates site of vascular leakage. (H) FITC-dextran leakage from the vasculature in the absence of CXCR3 blockade visualized on retinal flat mounts. Sites of vasculature breaks and leakage are indicated by white arrows. (I) Occludin staining on RPE flat mounts. Representative images taken from mid-retina. Breaks in occludin are indicated by white arrows. Scale bars, 100 μm and (I), 50 μm.

The receptor for CXCL9, CXCL10, and CXCL11 is CXCR3, and gene expression of *CXCR3* was strongly up-regulated in the eye with kinetics that mirrored T cell recruitment (fig. S8A). To determine whether immune cell infiltration of the retina involved CXCR3, we treated infected mice with a CXCR3 blocking antibody or an irrelevant antibody and assessed outcomes at PND15 (fig. S8B). Blockade of CXCR3 resulted in significantly more virus replicating in the lungs of infected mice but did not increase the amount of MCMV gene copies found in whole eyes (Fig. 6, A and B, and fig. S8C). However, histological analyses revealed significantly increased pp89 staining in the eye after CXCR3 blockade, with increases in the frequencies of pp89^+^ cells found in the RPE, ONL, and INL (fig. S8C and table S2). Thus, CXCR3 blockade altered viral control in the neuroretina despite the unchanged overall viral load. After CXCR3 blockade, we observed pp89^+^ puncta in microglia in the subretinal space (fig. S8C, red arrow) as well as in Müller glial cells traversing the retina (fig. S8C, yellow arrows), the latter of which was never seen in infected mice that were not treated with CXCR3 blocking antibody. These data suggest that blockade of CXCR3 increased the viral load in the neuroretina and that glial cells increased engulfment of viral debris in response. Unexpectedly, however, the pathology in the eyes of the αCXCR3-treated mice was almost completely rescued by CXCR3 blockade: There were no observed disruptions in the lamination of the retinal layers (Fig. 6C), nodules in the GCL were less frequent, and smaller (fig. S8, E and F), cellular infiltration of the neuroretina was essentially absent (Fig. 6, D and E, and fig. S8D) and cell death in the GCL was prevented (Fig. 6F and fig. S8H). In contrast, the choroids of mice treated with αCXCR3 still had an abundance of CD45^+^ immune cells, similar to the eyes of infected mice treated with an irrelevant antibody (Fig. 6, D and E, and fig. S8G), and exhibited elevated cell death (fig. S8I). Moreover, immune cells were clearly observed lining the retinal vasculature via flat mounts (outlined by GFAP^+^ astrocytes end feet) in αCXCR3-treated mice (Fig. 6E, white dotted lines). Thus, CXCR3 blockade completely prevented immune infiltration of the retina and any resulting retinal pathology but did not prevent immune cells from accumulating in the retinal vasculature and choroid.

### Prednisolone treatment also prevents retinal pathology after MCMV infection

These results from the CXCR3 blockade suggest that the immune response is the primary driver of retinal pathology after neonatal MCMV infection. As a second approach to test this idea, we dampened the immune response more generally by treating with the corticosteroid prednisolone (fig. S9A). Previous studies using this model showed that treatment with steroids rescued early MCMV-induced pathology in the brain (*29*). In our hands, prednisolone treatment led to increased viral titers in the lungs and eyes (fig. S9, B and C). Virus infected cells were found in all layers of the neuroretina as well as in the choroid (fig. S9D). Most infected mice that were treated with prednisolone died by PND15, but retinal pathology was clearly reduced by prednisolone treatment on PND10. Prednisolone treatment of infected mice led to normal lamination of retinal layers, the complete absence of rosette-like structures in the ONL, and a reduction in the nodules in the GCL (fig. S9E). Moreover, while an increased frequency of infected cells was detected in the prednisolone-treated eyes, the retinas were nearly devoid of CD45^+^ immune cells (fig. S9F), and cell death was reduced to baseline levels as assessed by TUNEL staining (fig. S9G). Last, glial cells appeared less activated: There was decreased microglia accumulation in the GCL (fig. S9H), decreased GFAP expression on astrocytes (fig. S9I), and no Müller glial cell activation (fig. S9I). Thus, immune suppression profoundly reverses retinal damage after neonatal infection by MCMV.

### BRB integrity is preserved by CXCR3 blockade

Since CXCR3 blockade prevented cellular infiltration of the retina after neonatal MCMV infection, we assessed the BRB integrity in these mice. Astrocytes are integral for BRB maintenance, and their end feet wrap around endothelial cells forming tight junctions that are involved in the formation of the blood-brain and blood-retinal barriers (*37*, *93*, *94*). For infected mice treated with irrelevant antibody, staining for GFAP^+^ astrocytes showed gaps in areas with cellular infiltration (white box), suggesting loss of astrocyte end feet and BRB leakage at the affected area (Fig. 6G). Strikingly, CXCR3 blockade completely prevented the formation of these astrocyte gaps, and hematopoietic cells remained trapped in the vasculature (outlined in white dotted lines) contained by the astrocyte end feet. Likewise, no leakage of FITC-dextran from the blood into the retina was observed in any of the CXCR3-blocked mice (Fig. 6H), and occludin staining of the outer BRB showed no loss of tight junction integrity, in contrast to the breakages observed in infected mice treated with an irrelevant antibody (Fig. 6I). These data show that CXCR3-blockade prevented the breakdown of BRB, thus preventing immune infiltration and subsequent retinal damage.

### CXCR3 blockade reduces glial activation after MCMV infection

**Fig. 7.**
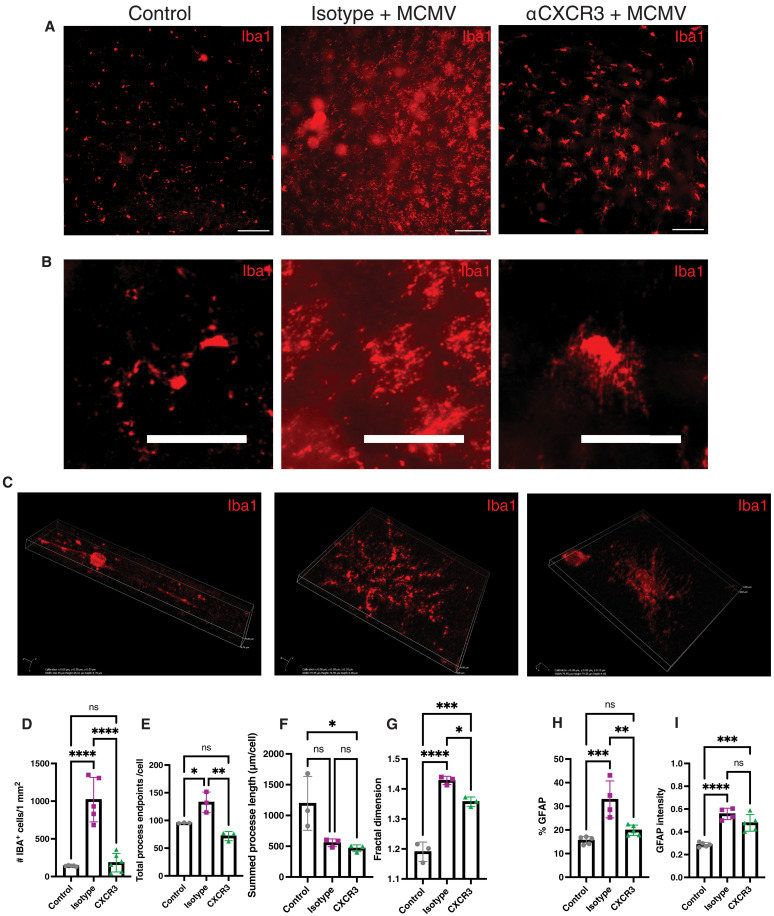
Blocking CXCR3 reduces glial cell activation. (**A**) Retinal flat mounts of control (uninfected) mice, and MCMV-infected mice treated with isotype control or αCXCR3 antibodies, were stained for Iba1^+^ cells (representative images from *n* = 5 to 8 per group). Scale bars, 100 μm (**B**) Magnified images of microglia from the groups in (A). Scale bars, 50 μm. (**C**) 3D projections of single microglia from the groups in (A). (**D**) Average Iba1^+^ cells per 1-mm^2^ area of DAPI. Counts were averaged from five retinal flat mount images per mouse from *n* = 5 mice per group. (**E** to **G**) Morphological analyses of Iba1^+^ microglia as assessed by (E) total process end points per cell. (F) Summed processes length per cell. (G) Cell complexity measured by fractal dimensions per cell. Each point shows an averaged value derived from *n* > 15 cells from an individual mouse and was graphed from *n* = 3 to 5 individual mice per analysis. Significance was analyzed by unpaired *t* test; *P < 0.05 **P < 0.01 ***P < 0.001 ****P < 0.0001. (**H** and **I**) Activation of astrocytes as assessed by (H) percent coverage of GFAP cells per DAPI area in each group and (I) intensity of GFAP staining in each group. Data show an average derived from five images per animal and *n* = 5 mice per group. Asterisks indicate statistical significance as in (D) to (G).

Microglia activation by MCMV was also significantly altered by CXCR3 blockade. Morphologically, αCXCR3 treatment led to microglia that had more, and thinner cell processes and appeared larger in size compared to microglia in infected mice not treated with the blocking antibody (Fig. 7, A to C). Quantifying the microglial numbers and morphology revealed that αCXCR3 treatment resulted in microglia with an intermediate activation phenotype. The CXCR3 blockade reversed the expansion of microglia numbers (Fig. 7G) and the increase in process end points per cell (Fig. 7H). Total branches and overall density of microglia from αCXCR3-treated mice were also significantly reduced but were still increased compared to uninfected control microglia (fig. S10, A and B), indicating some intermediate activation. Further, the summed process length and span ratio were still reduced regardless of CXCR3 blockade, indicating a more amoeboid shape (Fig. 7I and fig. S10C), and while the overall complexity of the cells measured by fractal dimensions was reduced by αCXCR3 treatment, the microglia in these mice were still significantly more complex than in uninfected controls (Fig. 7J). Microglia were less frequently found in the subretinal space in αCXCR3-treated eyes (fig. S10, D and E), suggesting less damage to the photoreceptors or RPE during αCXCR3 treatment. However, microglia found in the subretinal space still appeared to have pp89^+^ phagosomal–like inclusions in their cytoplasm (fig. S10F). Similarly, while CXCR3 blockade prevented the expansion of astrocytes (assessed by the percent coverage of GFAP-staining; Fig. 7K), the intensity of GFAP expression by astrocytes was unaffected by CXCR3 blockade (Fig. 7L). Notably, the up-regulation of GFAP on astrocytes in the GCL was also reduced in prednisolone-treated animals (fig. S9I). Together, these data suggest that CXCR3 blockade reduced and altered, but did not prevent, the activation of glial cells after MCMV infection.

### Blocking CXCR3 changes the expression of IFN-γ–stimulated genes in glial nodules

To assess transcriptional changes in response to CXCR3 blockade, we performed RNA-seq on isolated retinas and RPE/choroids from mice infected by MCMV (PND15) and treated with the αCXCR3-blocking antibody or irrelevant control antibody. As expected, MCMV infection drove significant differential gene expression in the retina and RPE/choroid compared to uninfected control tissues, regardless of CXCR3 blockade (fig. S11A). However, transcriptional differences between conditions were evident. To assess variations between samples and conditions and relate them to biological processes, GSVA for GO gene sets was used. Clustering analyses were performed to visualize sample variance and to cluster similar pathway enrichment into modules (fig. S11, B and C). For both retina and RPE/choroid samples, each condition clustered together. In the retina, αCXCR3-treated samples appeared to be generally more similar to uninfected controls than samples from infected mice treated with an irrelevant antibody (fig. S11B). However, there was some notable mouse-to-mouse variability, with two retina samples from mice treated with αCXCR3 (CX2 and CX3) that appeared more similar to infected mice treated with the irrelevant antibody. Even with this variability, αCXCR3 treatment strongly reverted genes in modules 4, 6, and 7 to a pattern that closely approximated uninfected retinas across all five samples in this group (fig. S11B). Module 4 pathways are mostly involved with metabolism, while modules 6 and 7 are involved with development. Furthermore, module 7 includes pathways involved in neuronal patterning. Thus, αCXCR3 treatment partially repairs the metabolic and developmental damage in the retina caused by MCMV infection. In contrast, αCXCR3 treatment had less of an impact on pathways in the RPE/choroid with the exception of module 3, which includes pathways involved with cell cycle and development (fig. S11C).

**Fig. 8.**
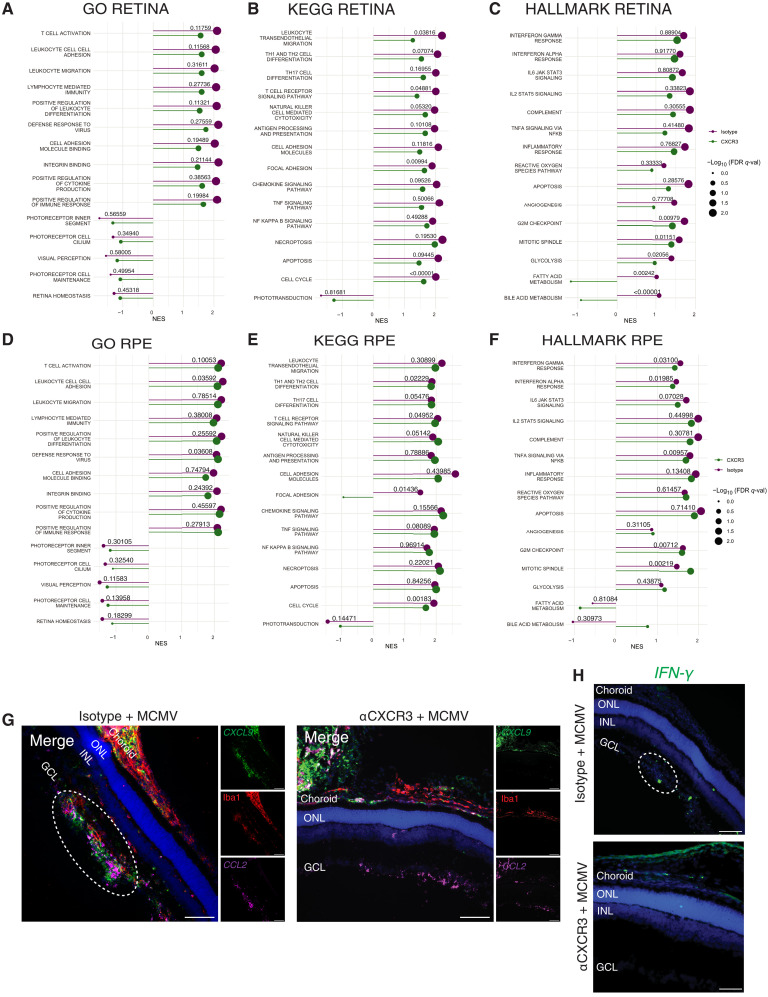
Blocking CXCR3 reduces many infection-induced transcriptional changes in the retina and inhibits glial cell chemokine production. (**A** to **F**) RNA-seq was performed on isolated retinas (*n* = 5) and RPE/choroids (*n* = 5) from control (uninfected) mice and MCMV-infected mice treated with isotype control or αCXCR3 antibodies. Differential genes were defined for each infected group relative to uninfected control samples. GSEA was used to identify enriched pathways for infected animals treated with isotype control or αCXCR3 antibodies. The highest relevant ranked enrichment was selected for each condition and plotted as lollipops by their NES (denoted as line length) and significance [−log_10_ (FDR *q* val), denoted as dot size]. Differences between the enrichment of pathways were determined by GSVA analysis, and *P* values were determined by unpaired *t* tests and are listed on each stem of the lollipop graphs. (A) Enrichment for GO for retinal tissue (MSibDB C5: GO gene set). (B) Enrichment for KEGG pathways for retinal tissue (MSibDB CP: KEGG). (C) Enrichment for Hallmark genes for retinal tissue (MSibDB H: Hallmark gene set). (D) Enrichment for GO for RPE tissue (MSibDB C5: GO gene set). (E) Enrichment for KEGG pathways for RPE tissue (MSibDB CP:KEGG). (F) Enrichment for Hallmark genes for RPE tissue (MSibDB H: Hallmark gene set). (**G** and **H**) In situ hybridization (RNAScope) of chemokine expression in eyes from MCMV-infected mice treated with isotype control or αCXCR3 antibodies. (G) Sections probed for *CXCL9* and *CCL2* transcripts and costained for Iba1^+^ cells. (H) Sections probed for *IFN-*γ transcripts. Representative of *n* = 3 mice per group. Scale bars, 100 μm.

To determine specific pathways that were affected by αCXCR3 treatment during MCMV infection, we used GSEA analyses of these samples. Immune response pathways were still significantly enriched in retinas from αCXCR3-treated mice but to a significantly reduced degree when compared to infected mice treated with irrelevant antibody (Fig. 8, A to C, and fig. S11D). Specifically, the αCXCR3 treatment significantly reduced the pathways involved in leukocyte transendothelial migration, T cell receptor signaling, focal adhesion, and natural killer (NK) cell–mediated cytotoxicity (Fig. 8, B and C, and fig. S11D). Notably, on the basis of our flat mount data (Fig. 6E), the residual enrichment of immune signatures is likely attributable to immune cells contained in the inner retinal vasculature and ciliary body, which could not be removed. In addition, CXCR3-blockade led to significantly reduced enrichment of cell cycle and metabolic pathways compared to retinas treated with an irrelevant antibody, together supporting the conclusion that αCXCR3 treatment partially restored normal retinal development (Fig. 8C and fig. S11D). The isolation of the retina in these experiments allowed us to detect significant negative enrichment of pathways involved with visual perception (Fig. 8A), indicating that visual function pathways were indeed disrupted by MCMV infection as was suggested after whole-eye RNA-seq (Fig. 3B). However, CXCR3 blockade did not significantly change these pathways, suggesting that viral infection may still cause some retinal function defects.

In the RPE/choroid, immune-related pathways were similarly enriched regardless of αCXCR3 treatment with the exception of some small but significant differences detected in cytokine, chemokine, and T cell response pathways (Fig. 8, D to F, and fig. S11E). Together, these data suggest that an immune response is still occurring in the RPE/choroid regardless of treatment but to a lesser degree in mice treated with αCXCR3.

Last, we assessed retinal glial nodules specifically via RNAscope in situ hybridization and immune fluorescence. Strikingly, glial nodules and, indeed, most of the neuroretina expressed no *CXCL9*, *CXCL10*, *CXCL11*, iNOS, or MHC-II (Fig. 8G and fig. S12, A to E). To quantify this, we measured the colocalization of microglia with *CXCL9*, iNOS, and MHC-II across the whole retina, regardless of whether cells localized to a nodule. These analyses showed that while ~15% of all microglia expressed MHC-II and iNOS on PND15, this was entirely ablated after CXCR3 blockade (fig. S12, C to E). Likewise, *IFN-*γ was absent from the neuroretina in αCXCR3-treated mice, while *CCL2*, which can be produced by astrocytes in direct response to CMV (*90*), was unaffected (Fig. 8, G and H). Together, these data suggest that blocking CXCR3 prevented the expression of IFN-γ–stimulated genes in retinal glial nodules, presumably as a result of preventing lymphocyte infiltration, altering the outcomes of immune response to MCMV infection in the retina. These data show that immune interventions were able to preserve the BRBs and prevent retinal pathology while partially reverting glial cell activation and restoring retinal development during neonatal MCMV infection.

## DISCUSSION

Approximately 20% of children with symptomatic congenital CMV infection develop severe vision impairment (*3*–*6*, *33*). However, the mechanisms of disease are unknown, and no prior animal models have been developed. Our data demonstrate that MCMV infection of newborn mice drove profound, focal pathology in developing retinas, similar to what is observed in humans. Strikingly, using this model, we found that by targeting the immune system, most disease manifestations were reduced. HCMV and MCMV are both well-known to cause severe retinitis in immune-suppressed hosts. HCMV retinitis is a common complication of T cell decline in patients with AIDS, and extensive work in the MCMV model has clearly demonstrated that T cells are protective against severe retinitis in adults (*50*, *51*, *95*). In contrast, while newborn mice are immune immature, they do mount MCMV-specific T cell responses with slightly delayed kinetics (*96*), and T cells are critical for survival of infected pups (*24*, *26*, *27*). Additional studies have attempted to correlate the development of symptoms after congenital infection with T cell responses in the infant but without conclusive results (*97*–*99*). In our model, the arrival of T cells in the eyes of mice corresponded to a reduction in detectable MCMV-infected cells in the eye, and both prednisolone treatment and CXCR3 blockade increased the numbers of pp89^+^ cells we could find in the retina. Thus, we might have expected that blocking T cell responses with prednisolone or T cell migration with αCXCR3 would lead to more pathology, mirroring an immune-deficient state. Unexpectedly however, the opposite was true: Immune suppression preserved the BRB integrity, inhibited retinal cell death, reduced the size of glial nodules, prevented glial cells from expressing IFN-γ–stimulated genes that are normally associated with neurotoxicity, and restored many features of normal retinal development and metabolism. Thus, at least transiently, immune suppression blocked MCMV-driven retinitis during eye development. These data suggest that the timing and location of T cell responses may be critical. If T cells respond rapidly enough to restrict infection of neural tissues, this could be protective. In contrast, delayed T cell responses would allow viral invasion of neural tissues with subsequent immune responses within these tissues leading to pathology.

Previous work has used this model to assess aging-related damage to the retina (*58*), but these investigators did not describe any notable neonatal pathology or investigate early life immune responses. The focal nature of the observed pathology in the eye meant that multiple serial sections or whole retinal flat mounts were necessary to find the affected regions in every mouse. Nevertheless, pathology was evident in all infected mice, and our data, with and without immune suppression, allowed us to develop a temporal model of this neonatal MCMV retinitis. MCMV loads were below our limit of detection on PND5, but the mice were beginning to make an innate immune response as assessed by RNA-seq, suggesting that viral amplification elsewhere in the body led to viremia in the first week that seeded the eye with MCMV. This had the effect of strongly activating retinal glial cells, including marked activation of astrocytes and microglia by PND10 and the development of nascent glial nodules in the GCL before the T cells arrived in the retina. Microglia normally reside in the inner and outer plexiform areas but migrate toward damage (*68*, *70*–*72*, *100*). On the basis of our data and prior work from Lokensgard and colleagues (*90*), we speculate that activated microglia migrated toward the CCL2 expressed by activated astrocytes in the GCL to initiate nodule formation. Such nodules may be a common feature of CNS infections by CMV and have been seen in the brains of CMV-infected mice (*11*, *28*, *86*), guinea pigs (*82*–*85*), and humans (*87*). Notably, CMVs encode virally derived chemokines that attract myeloid cells. Although it is unknown whether microglia can respond to these viral chemokines, it is possible that they might also contribute to the formation of glial nodules. Future work is needed to determine whether the MCMV-derived chemokine MCK2 contributes to retinal pathology in our model.

Next, T cells were recruited to the eye and accumulated in the growing glial nodules. Together, these cells formed a unique aberration in the GCL composed of astrocytes, microglia, infiltrating CD8^+^ T cells, and activated Müller glial cells (Fig. 5 and movie S1 for a three-dimensional rendering of a nodule). These nodules appeared to create a barrier of astrocytes around an area filled with CD8^+^ T cells and microglia, with the microglia expressing T cell attracting chemokines and a neurotoxic phenotype. Of note, cell death was particularly enriched in these nodules, as were microglia containing pp89^+^ puncta in their cytoplasm. Thus, we propose these nodules as potential sites for the killing and clearance of virally infected cells. Previous studies have described this phenomenon in other models of retinal disease and have suggested that their purpose is to encircle an area of damage, shielding the surrounding area from further destruction (*101*, *102*).

On the basis of the timing of these events, we hypothesized that the CXCR3 blockade would limit the recruitment of CD8 T cells to the glial nodules within the retina but not prevent T cells from gaining access to the retina per se. Strikingly, however, blocking this receptor completely prevented T cells from accessing the retina, trapping them in the retinal vasculature, and prevented most of the pathological outcomes. These data strongly suggest that CXCR3 is used by T cells first within the vasculature, resulting in the breakdown of the BRBs. Future work will be needed to define the specificity/function of the T cells responsible and to determine whether T cell recognition of antigen in the vasculature is necessary for disruption of the BRBs. Intravascular staining cannot be used to localize antigen-specific T cells since the BRB becomes leaky. Thus, addressing these unknowns will likely require transgenic T cells that can be identified histologically after transfer, and model systems that can define the cells presenting viral antigens (e.g., Cre-Lox systems deleting MHC from specific cell types). In addition, given the small numbers of T cells in the neonatal eye, single-cell RNA-seq may allow more precise characterization.

Regardless of the precise role of T cells in pathology, blockade of CXCR3 not only prevented lymphocyte invasion of the retina but also prevented the microglia and astrocytes from progressing beyond the initial activation observed at PND10. In infected mice treated with CXCR3 blockade, the microglia were smaller and more complex than resting microglia and they were still found in the GCL, but their morphology was not the same as in MCMV-infected mice treated with isotype control antibodies. Moreover, their numbers were restored to baseline (uninfected) levels by the CXCR3 blockade. Similarly, astrocytes still appeared activated on the basis of the intensity of GFAP staining and their expression of CCL2, but their numbers were restored to baseline levels by CXCR3 blockade. Thus, MCMV likely directly activated these cells, but the arrival of immune cells caused the nodules to progress to the enlarged, destructive state with the central, T cell–rich killing zone containing neurotoxic microglia, surrounded by activated astrocytes and Müller glia. Notably, the activation of Müller glia was prevented by prednisolone and CXCR3 blockade, suggesting that Müller glial cells are not directly activated by MCMV at this stage but depend on the progression of the immune response.

CXCR3 blockade also resulted in a distinct absence of the chemokines CXCL9, CXCL10, and CXCL11, as well as iNOS and MHC-II in microglia, indicating an absence of neurotoxic glial cells. These genes are known to be induced by IFN-γ (*80*, *81*, *91*, *92*). Thus, loss of BRB integrity and secretion of IFN-γ in the retina likely plays a central role in the progression of the glial nodules and the neurotoxic phenotype. Recent work has suggested that NK cells are a critical early source of IFN-γ in the brain in the cMCMV model (*23*). In this work, the NK cells arrived by PND6 and peaked on PND8, driving early IFN-γ–dependent microglial activation. We have stained for NK cells but rarely find these cells in the retina. Moreover, immune infiltration of any kind was still absent from the retina as late as PND10, suggesting that these early NK cell responses are not involved in retinal pathology. Thus, we propose that T cell–derived IFN-γ may be more critical in the retina, although this requires future study. In our hands, depleting NK cells in MCMV-infected pups resulted in mortality before PND15, precluding a direct test of the role of NK cells on late retinal pathology and glial activation. Overall, these data describe a step-wise induction of immune pathology during MCMV retinal infection in neonatal mice and show that targeting immune response mechanisms can ameliorate disease, at least transiently.

Although there are limitations in comparing a mouse pathogen to its human counterpart, our data suggest many parallels between this mouse model and the human disease. In humans, congenital infection leads to focal chorioretinitis and optic neuritis (*4*, *33*, *34*). In our model, we observed profound focal immune infiltration of the retina and optic nerve. These focal patterns observed during human infection tended to follow the vasculature and have been described as frosted branch angiitis of inflamed blood vessels (*34*–*36*). Likewise, in our mouse model, the immune cells tended to cluster around blood vessels in areas with disrupted BRBs. Thus, this cMCMV model appears to replicate the human congenital disease.

The current standard for treatment for congenital CMV infection is the antiviral drug ganciclovir. While this treatment can rapidly resolve active viral replication, it would have only an indirect impact on immune-mediated disease. Thus, targeting the immune system could be a critical adjunct therapy to any virus-targeted interventions. In the retina, prednisone treatment has been used effectively to treat retinal necrosis caused by different herpesviruses, including CMV-driven retinitis in adults (*103*). Others have shown in guinea pigs or mouse models of disease caused by congenital CMV infection that immune suppression reduced inner ear pathology (*29*, *104*). Our data using αCXCR3 as a treatment demonstrated that blocking lymphocyte recruitment prevented the observed pathology and restored retinal development. A recent report suggested that blocking CXCR3 may also alleviate T cell–driven pulmonary pathology caused by influenza infection (*105*). Despite these successes in mice, we suspect that inhibition of T cell migration is unlikely to be a viable clinical approach for congenital CMV infection as it would depend on catching the disease before T cells have reached the eye. This may not be possible if T cells infiltrate the retina before birth or diagnosis. However, our data further suggest that IFN-γ–stimulated genes were absent from glial cells when lymphocyte entry was blocked. Microglia are known to be driven toward a neurotoxic state by IFN-γ (*80*), and, indeed, we observed iNOS and MHC-II, along with *CXCL9*, *10*, and *11* expression in microglia from infected eyes but not when CXCR3 was blocked. Thus, IFN-γ could be a critical central mediator of retinal pathology, as has also been proposed for the brain (*23*). While this question awaits future studies, it is important to note that T cell and NK cell responses are vital for MCMV control, including in this neonatal infection model (*24*). Therefore, therapies targeting T cell or NK cell effector functions will almost certainly inhibit viral control and promote disease over time. Blocking CXCR3 resulted in increased viral loads in the lungs and increased viral staining in the eyes. It is our view that immune targeting would have to be combined with antiviral therapy to limit both viral growth and immunopathology.

One main job of glial cells in the eye and brain is to engulf and recycle damaged cells, and we found clear evidence of microglia containing pp89-stained puncta in their cytoplasm in the vicinity of infected cells. Moreover, both microglia and Müller glial cells appeared to participate in the engulfment of viral debris after CXCR3 blockade. This raises the intriguing possibility that glial cells may be participating in the control of viral infection in the neuroretina and suppressing viral loads in the absence of T cells.

Transcriptional evaluation allowed us to begin defining how CMV and the associated immune response disrupted normal eye development. The data suggest that infection-associated pathology affected metabolism, cell migration, differentiation, and neuronal patterning. Likewise, assessing isolated retinas at PND15 showed a negative enrichment for pathways involved in visual function. Further work will be needed to determine how these developmental and metabolic changes affect visual perception. While it was apparent that αCXCR3 treatment markedly improved the retinal inflammation and normalized many of the developmental and metabolic pathways in the retina, it was less clear that αCXCR3 treatment significantly restored gene pathways associated with visual processes. Thus, further work will be needed to determine what effect these early-life disruptions, or the immune interventions, have on long-term visual function as the mice grow into adults.

In summary, this study has characterized MCMV infection of the eye during development and implicated the immune response in disease manifestations. Since newborn mice are at a similar developmental stage as a late second-trimester human fetus—a time when CMV is known to cross the placenta—this model allowed us to explore potential developmental changes caused by the CMV in the eye. By blocking lymphocyte recruitment via chemokine receptor CXCR3, we prevented many of the pathological outcomes of MCMV infection. Thus, our study may suggest previously unidentified therapeutic targets to prevent CMV-induced retinal pathology in newborns.

## MATERIALS AND METHODS

### Study design

The objective of this study was to determine the pathogenesis of cCMV infections of the retina. We used the established cMCMV animal model and assessed eye development, pathology, viral loads, and immune responses via (i) frozen section and flat mount histology, (ii) live ocular imaging, (iii) transcriptional analyses, and (iv) viral plaque assays and quantitative polymerase chain reaction (qPCR). In addition, we determined whether interference with immune responses would alter the outcomes using blocking antibodies or prednisolone treatment of the mice. Breeder pairs were never reused after a litter was infected with MCMV. Each litter was used for a single experimental condition, without exclusions. At least two independent liters were used for each experimental end point. For all analyses, we examined at least three randomly chosen individual mice (technical replicates) from two to three litters (biological replicates). Time points for analyses were chosen by pilot experiments to define the kinetics of retinal infection. All animals were housed in the same facility with 12-hour day/night cycles. Data were not blinded, and no data were excluded from this study.

### Animals and viruses

All experiments were approved by the Thomas Jefferson University Institutional Animal Care and Use Committee (approved protocol number 01209). For all experiments, newborn BALB/c mice were derived from matings of 6- to 7-week-old BALB/c mice purchased from the Jackson Laboratory (strain number 000651). Stocks of the K181 strain of MCMV (GenBank AM886412.1, originally provided by E. Mocarski) were produced from M2-10B4 cells (ATCC, CRL-1972) and quantified by plaque assay as previously described (*106*). Newborn BALB/c pups were inoculated intraperitoneally with 200 PFU in 20 μl of PBS less than 24 hours after birth. Control groups were injected with an equal volume of PBS alone. To track weight gain, mice in a litter were weighed together as a group and the total weight divided by the number of pups to establish an average pup weight per litter. The mice were euthanized at PND5, 10, 15, and 28 as described in the figure legends, and the eye and lung tissues were collected without perfusion for further analyses. Virus titers in lung and eye tissue were determined by plaque assay (*106*) or by qPCR for the MCMV-E1 gene as described previously (*107*). In brief, viral DNA was recovered with the Allprep DNA/RNA mini kit (Qiagen), and qPCR assay was conducted with the TaqMan Fast Universal Master Mix using the following primers and probe: Primer E1 for: TCGCCCATCGTTTCGAGA; Primer E1 rev: TCTCGTAGGTCCACTGACGGA; Probe: ACTCGAGTCGGACGCTGCATCAGAAT.

### Prednisolone treatments

For prednisolone experiments, infected or control mice were treated with prednisolone sodium phosphate starting on PND4 by daily injection (7 mg/kg, ip) in 50 μl of PBS, or PBS as a control, as previously described (*29*). Prednisolone treatment concluded on PND9, and all mice were euthanized the following day (PND10).

### CXCR3 blockade

To block CXCR3, the mice were treated by injection with anti-CXCR3 antibody (25 mg/kg, ip, clone CXCR3-173) or an irrelevant isotype control antibody (clone PIP), both purchased from BioXCell. Antibody treatments began on PND0 (at the time of infection or PBS injection) and were repeated on PND5 and PND10. All mice were euthanized on PND15.

### Live ocular imaging

Confocal SLO and spectral domain OCT (SD-OCT) were used to visualize ocular structures of MCMV-infected 1-month-old mice. The mice were weighed individually and anesthetized with a mixture of ketamine (100 mg/kg) and xylazine (10 mg/kg). To anesthetize the eyes, the mice were given a drop of 0.5% proparacaine hydrochloride ophthalmic solution, followed by pupil dilation with a drop of 1% tropicamide solution. The Spectralis HRA + OCT (Heidelberg Engineering) was used to obtain infrared reflectance, autofluorescence images of the retina and RPE, and SD-OCT images of the ocular structures.

### H&E, immunofluorescence, TUNEL staining, and quantification

Eyes were enucleated and placed immediately in plastic molds, covered in NEG50 solution (Epredia), and then flash frozen in liquid nitrogen for cryopreservation. Serial cryosections (10 μm) of the eye were collected starting at ~one-fourth of the way into the globe and were continuously collected through ~500 μm of tissue until passing the optic nerve. Sections were placed on slides and stored at −20°C. H&E staining was performed by the Thomas Jefferson University, Translational Research/Pathology core facility. To perform immunofluorescence on thin sections, the tissue was fixed with either cold acetone or 4% paraformaldehyde (PFA) for 10 min and then blocked and permeabilized with 3% bovine serum albumin (BSA) and 0.1% Triton X-100 for 1 hour. After blocking, primary antibodies (table S4) were diluted in blocking buffer and incubated for either 1 hour at room temperature or overnight at 4°C, followed by the appropriate secondary antibody (table S4) for 30 min at room temperature. Last, slides were stained with 4′,6-diamidino-2-phenylindole (DAPI, 1:100) for 10 min and mounted under cover glass with Flouromount-G mounting medium. Between each step described, the slides were washed three times with 1× PBS. For detection of MCMV-infected cells, the sections were stained with pp89 (IE1)–specific antibody clone 6/58/1 (*108*). In brief, the antibody was produced in a bioreactor and concentrated and purified by Protein A/G (NAB Protein A/G spin kit, Thermo Fisher Scientific) as per the manufacturer’s protocol. Antibody titer was determined using Easy Titer Mouse IgG Assay Kit (Thermo Fisher Scientific) and labeled with Alexa Fluor 488 antibody labeling kit (Thermo Fisher Scientific), following the manufacturer’s protocol. TUNEL staining to detect cell death was preformed using ApopTag Fluorescein In situ Apoptosis Detection Kit (Millipore Sigma) following the manufacturer’s protocol.

### Whole retina and RPE/choroid flat mounts

For whole mounts, the eyes were enucleated and a small incision was made in cornea before immersion in 4% PFA for 8 min. After fixation, the cornea and lens were removed under a dissection microscope, and the retina was separated from the RPE/choroid. Both tissues were immersed in 4% PFA for an additional 8 min. The tissue was then washed with 1× PBS, permeabilized with 0.3% Triton X-100, and blocked with 5% BSA + 0.1% Triton X-100 for 1.5 hours. For staining, the tissue was incubated in 96-well trays overnight with primary antibodies (table S4) at 4°C, followed by secondary antibodies and DAPI. As above, the tissues were washed three times with 1× PBS between each step. After staining, the retina and RPE/choroid were placed on a glass slide and cut radially to flatten the tissue before adding Flouromount-G mounting medium and a coverslip.

### Image capture and quantifiction

All H&E images were captured with Nikon Eclipse Ci. Immunofluorescent images were captured with either a Leica DM5000 B or Nikon Eclipse Ci for transmitted light or a Nikon A1R confocal microscope for confocal images. Quantification of immunofluorescence was determined with ImageJ as described in the figure legends.

### Assessment of BRB integrity

To assess the integrity of the BRB, infected or control mice were injected with FITC-dextran (10,000 Da, Invitrogen). For this, the mice were anesthetized with isoflurane and FITC-dextran was injected retro-orbitally. The mice were euthanized 5 min after the injection, and the eyes were enucleated and immediately frozen in NEG50.

### Microglia morphological analysis

Retinal flat mounts stained with Iba1 were used for morphological analysis. All cell morphology analysis was preformed using ImageJ skeleton and fractal analyses as previously described (*109*) using five images per animal for skeleton analyses and 15 cells per animal for fractal analyses.

### RNA in situ hybridization (RNAscope)

RNAscope (ACDBio) in situ hybridization was performed using the Multiplex Fluorescent Reagent Kit v2. Samples were prepared following the manufacturer’s fresh-frozen sample preparation and pretreatment protocol with some adjustments as follows: (i) The PFA fixation time was increased from 15 to 30 min, and (ii) the slides were baked at 37°C for 30 min after the hydrogen peroxide step. Probes were purchased from ACDBio to visualize CXCL9, CXCL10, CXCL11, CCL2, and IFN-γ. Opal dyes (Akoya Biosciences) were used as secondary staining. RNAscope was combined with Immunofluorescence (table S4) using the protocol provided by ACD.

### RNA sequencing

For analyses of whole eyes over time after infection, the eyes were homogenized using a bead beater, and RNA was extracted using DNA/RNA extraction kit (Allprep DNA/RNA mini kit, Quiagen). Triplicate samples were used for analyses, each consisting of equal amounts of RNA combined from two eyes derived from two different pups from the same condition. To select pups for each pair, we chose mice with higher and lower viral titers to normalize any mouse-to-mouse variations in viral loads affecting the data. For analyses of eyes after CXCR3 blockade, the retinas and RPE/choroids were separated under a dissection microscope and homogenized using a pestle in a 1.5-ml conical tube. RNA was extracted as above, and sequencing was performed on matched retinas and RPE/choroids (i.e., derived from the same eyes) from five individual mice per group. In all cases, sequencing, alignment, quality control, and differential analysis (DeSeq2) was performed by Novogene. Clustering of DEGs and plotting were performed as previously described (*110*). Go enrichment analyses (g:Profiler) were performed against gene list derived from clustering analysis. Additional GSEA analyses were performed using GSEA_4.2.3 Mac App (Broad Institute) against GO gene sets, Hallmark gene sets, KEGG pathways, and curated C8 cell type signature gene sets (Molecular Signatures Database). Variance between all samples sequenced against curated gene sets was performed using GSVA (*111*). All analyses were visualized using ggplot R packages. Further specifics on individual comparisons are described in figure legends.

### Statistical analysis

Statistical analyses of RNA-seq data were conducted using DeSeq2. DEGs were defined as genes with padj <0.1. Significance of gene set enrichment was determined by unpaired *t* test comparing individual variance scores from GSVA. All other statistical analyses were performed using GraphPad Prism version 10.0.0. Significance was determined using unpaired *t* test and one-way analysis of variance (ANOVA) with multiple comparisons as described in figure legends.

## References

[1] A. Kenneson, M. J. Cannon, Review and meta-analysis of the epidemiology of congenital cytomegalovirus (CMV) infection. Rev. Med. Virol. 17, 253–276 (2007). 17579921 10.1002/rmv.535

[2] M. C.-J. Cheeran, J. R. Lokensgard, M. R. Schleiss, Neuropathogenesis of congenital cytomegalovirus infection: Disease mechanisms and prospects for intervention. Clin. Microbiol. Rev. 22, 99–126 (2009). 19136436 10.1128/CMR.00023-08 PMC2620634

[3] S. B. Boppana, R. F. Pass, W. J. Britt, S. Stagno, C. A. Alford, Symptomatic congenital cytomegalovirus infection neonatal morbidity and mortality. Pediatr. Infect. Dis. J. 11, 93–98 (1992). 1311066 10.1097/00006454-199202000-00007

[4] D. K. Coats, G. J. Demmler, E. A. Paysse, L. T. Du, C. Libby, Ophthalmologic findings in children with congenital cytomegalovirus infection. J. AAPOS 4, 110–116 (2000). 10773810 10.1067/mpa.2000.103870

[5] H. D. Jin, G. J. Demmler-Harrison, D. K. Coats, E. A. Paysse, A. Bhatt, J. C. Edmond, K. G. Yen, P. Steinkuller, J. Miller, Long-term visual and ocular sequelae in patients with congenital cytomegalovirus infection. Pediatr. Infect. Dis. J. 36, 877–882 (2017). 28399055 10.1097/INF.0000000000001599 PMC5555772

[6] S. Boppana, C. Amos, W. Britt, S. Stagno, C. Alford, R. Pass, Late onset and reactivation of chorioretinitis in children with congenital cytomegalovirus infection. Pediatr. Infect. Dis. J. 13, 1139–1142 (1994). 7892084 10.1097/00006454-199412000-00012

[7] K. B. Fowler, F. P. McCollister, A. J. Dahle, S. Boppana, W. J. Britt, R. F. Pass, Progressive and fluctuating sensorineural hearing loss in children with asymptomatic congenital cytomegalovirus infection. J. Pediatr. 130, 624–630 (1997). 9108862 10.1016/s0022-3476(97)70248-8

[8] S. Prasad, S. Hu, W. S. Sheng, P. Chauhan, A. Singh, J. R. Lokensgard, The PD-1: PD-L1 pathway promotes development of brain-resident memory T cells following acute viral encephalitis. J. Neuroinflammation 14, 82 (2017). 28407741 10.1186/s12974-017-0860-3 PMC5390367

[9] P. Chauhan, S. Hu, W. S. Sheng, S. Prasad, J. R. Lokensgard, Modulation of microglial cell Fcγ receptor expression following viral brain infection. Sci. Rep. 7, 41889 (2017). 28165503 10.1038/srep41889 PMC5292951

[10] J. R. Lokensgard, M. B. Mutnal, S. Prasad, W. Sheng, S. Hu, Glial cell activation, recruitment, and survival of B-lineage cells following MCMV brain infection. J. Neuroinflammation 13, 114 (2016). 27207308 10.1186/s12974-016-0582-y PMC4874004

[11] J. R. Lokensgard, S. J. Schachtele, M. B. Mutnal, W. S. Sheng, S. Prasad, S. Hu, Chronic reactive gliosis following regulatory T cell depletion during acute MCMV encephalitis. Glia 63, 1982–1996 (2015). 26041050 10.1002/glia.22868 PMC4670295

[12] S. J. Schachtele, S. Hu, W. S. Sheng, M. B. Mutnal, J. R. Lokensgard, Glial cells suppress postencephalitic CD8^+^ T lymphocytes through PD-L1. Glia 62, 1582–1594 (2014). 24890099 10.1002/glia.22701 PMC4141010

[13] M. B. Mutnal, S. Hu, J. R. Lokensgard, Persistent humoral immune responses in the CNS limit recovery of reactivated murine cytomegalovirus. PLOS ONE 7, e33143 (2012). 22412996 10.1371/journal.pone.0033143 PMC3295797

[14] S. J. Schachtele, M. B. Mutnal, M. R. Schleiss, J. R. Lokensgard, Cytomegalovirus-induced sensorineural hearing loss with persistent cochlear inflammation in neonatal mice. J. Neurovirol. 17, 201–211 (2011). 21416394 10.1007/s13365-011-0024-7 PMC3098308

[15] M. B. Mutnal, M. C.-J. Cheeran, S. Hu, J. R. Lokensgard, Murine cytomegalovirus infection of neural stem cells alters neurogenesis in the developing brain. PLOS ONE 6, e16211 (2011). 21249143 10.1371/journal.pone.0016211 PMC3020957

[16] M. B. Mutnal, S. Hu, M. R. Little, J. R. Lokensgard, Memory T cells persisting in the brain following MCMV infection induce long-term microglial activation via interferon-γ. J. Neurovirol. 17, 424–437 (2011). 21800103 10.1007/s13365-011-0042-5 PMC3204167

[17] M. B. Mutnal, M. C.-J. Cheeran, S. Hu, M. R. Little, J. R. Lokensgard, Excess neutrophil infiltration during cytomegalovirus brain infection of interleukin-10-deficient mice. J. Neuroimmunol. 227, 101–110 (2010). 20655600 10.1016/j.jneuroim.2010.06.020 PMC2942961

[18] M. C.-J. Cheeran, M. B. Mutnal, S. Hu, A. Armien, J. R. Lokensgard, Reduced lymphocyte infiltration during cytomegalovirus brain infection of interleukin-10–deficient mice. J. Neurovirol. 15, 334–342 (2009). 19626525 10.1080/13550280903062797 PMC2888792

[19] M. C.-J. Cheeran, G. Gekker, S. Hu, J. M. Palmquist, J. R. Lokensgard, T cell–mediated restriction of intracerebral murine cytomegalovirus infection displays dependence upon perforin but not interferon-γ. J. Neurovirol. 11, 274–280 (2005). 16036807 10.1080/13550280590952808 PMC7095405

[20] M. C.-J. Cheeran, G. Gekker, S. Hu, X. Min, D. Cox, J. R. Lokensgard, Intracerebral infection with murine cytomegalovirus induces CXCL10 and is restricted by adoptive transfer of splenocytes. J. Neurovirol. 10, 152–162 (2004). 15204920 10.1080/13550280490441130

[21] Y.-P. Zhou, M.-J. Mei, X.-Z. Wang, S.-N. Huang, L. Chen, M. Zhang, X.-Y. Li, H.-B. Qin, X. Dong, S. Cheng, L. Wen, B. Yang, X.-F. An, A.-D. He, B. Zhang, W.-B. Zeng, X.-J. Li, Y. Lu, H.-C. Li, H. Li, W.-G. Zou, A. J. Redwood, S. Rayner, H. Cheng, M. A. McVoy, Q. Tang, W. J. Britt, X. Zhou, X. Jiang, M.-H. Luo, A congenital CMV infection model for follow-up studies of neurodevelopmental disorders, neuroimaging abnormalities, and treatment. JCI Insight 7, e152551 (2022). 35014624 10.1172/jci.insight.152551 PMC8765053

[22] R. D. Bradford, Y.-G. Yoo, M. Golemac, E. P. Pugel, S. Jonjic, W. J. Britt, Murine CMV-induced hearing loss is associated with inner ear inflammation and loss of spiral ganglia neurons. PLOS Pathog. 11, e1004774 (2015). 25875183 10.1371/journal.ppat.1004774 PMC4395355

[23] D. Kveštak, V. J. Lisnić, B. Lisnić, J. Tomac, M. Golemac, I. Brizić, D. Indenbirken, M. C. Brdovčak, G. Bernardini, F. Krstanović, C. Rožmanić, A. Grundhoff, A. Krmpotić, W. J. Britt, S. Jonjić, NK/ILC1 cells mediate neuroinflammation and brain pathology following congenital CMV infection. J. Exp. Med. 218, e20201503 (2021). 33630019 10.1084/jem.20201503 PMC7918636

[24] G. R. B. Bantug, D. Cekinovic, R. Bradford, T. Koontz, S. Jonjic, W. J. Britt, CD8^+^ T lymphocytes control murine cytomegalovirus replication in the central nervous system of newborn animals. J. Immunol. 181, 2111–2123 (2008). 18641350 10.4049/jimmunol.181.3.2111 PMC4161464

[25] M. C. Seleme, K. Kosmac, S. Jonjic, W. J. Britt, Tumor necrosis factor alpha-induced recruitment of inflammatory mononuclear cells leads to inflammation and altered brain development in murine cytomegalovirus-infected newborn mice. J. Virol. 91, e0198316 (2017). 10.1128/JVI.01983-16 PMC5375689 28122986

[26] I. Brizić, L. Hiršl, M. Šustić, M. Golemac, W. J. Britt, A. Krmpotić, S. Jonjić, CD4 T cells are required for maintenance of CD8 TRM cells and virus control in the brain of MCMV-infected newborn mice. Med. Microbiol. Immunol. 208, 487–494 (2019). 30923899 10.1007/s00430-019-00601-0 PMC6640853

[27] I. Brizić, B. Šušak, M. Arapović, P. C. Huszthy, L. Hiršl, D. Kveštak, V. Juranić Lisnić, M. Golemac, E. Pernjak Pugel, J. Tomac, A. Oxenius, W. J. Britt, J. Arapović, A. Krmpotić, S. Jonjić, Brain-resident memory CD8^+^ T cells induced by congenital CMV infection prevent brain pathology and virus reactivation. Eur. J. Immunol. 48, 950–964 (2018). 29500823 10.1002/eji.201847526 PMC6422351

[28] Đ. Cekinović, M. Golemac, E. P. Pugel, J. Tomac, L. ČiČin-Šain, I. Slavuljica, R. Bradford, S. Misch, T. H. Winkler, M. Mach, W. J. Britt, S. Jonjić, Passive immunization reduces murine cytomegalovirus-induced brain pathology in newborn mice. J. Virol. 82, 12172–12180 (2008). 18842707 10.1128/JVI.01214-08 PMC2593357

[29] K. Kosmac, G. R. Bantug, E. P. Pugel, D. Cekinovic, S. Jonjic, W. J. Britt, Glucocortiocoid treatment of MCMV infected newborn mice attenuates CNS inflammation and limits deficits in cerebellar development. PLOS Pathog. 9, e1003200 (2013). 23505367 10.1371/journal.ppat.1003200 PMC3591306

[30] K. Ikuta, H. Ogawa, H. Hashimoto, W. Okano, A. Tani, E. Sato, I. Kosugi, T. Kobayashi, K. Omori, T. Suzutani, Restricted infection of murine cytomegalovirus (MCMV) in neonatal mice with MCMV-induced sensorineural hearing loss. J. Clin. Virol. 69, 138–145 (2015); https://doi.org/10.1016/j.jcv.2015.06.083. 26209396 10.1016/j.jcv.2015.06.083

[31] T. Koontz, M. Bralic, J. Tomac, E. Pernjak-Pugel, G. Bantug, S. Jonjic, W. J. Britt, Altered development of the brain after focal herpesvirus infection of the central nervous system. J. Exp. Med. 205, 423–435 (2008). 18268036 10.1084/jem.20071489 PMC2271002

[32] B. Lisnić, J. Tomac, D. Cekinović, S. Jonjić, V. Juranić Lisnić, Rodent Models of Congenital Cytomegalovirus Infection in *Methods in Molecular Biology*, (Humana Press Inc., 2021), vol. 2244, pp. 365–401. 10.1007/978-1-0716-1111-1_18 33555596

[33] S. Ghekiere, K. Allegaert, V. Cossey, M. Van Ranst, C. Cassiman, I. Casteels, Ophthalmological findings in congenital cytomegalovirus infection: When to screen, When to treat? J. Pediatr. Ophthalmol. Strabismus 49, 274–282 (2012). 22800795 10.3928/01913913-20120710-03

[34] F. M. Recchia, Treatment of congenital cytomegalovirus retinitis with intravitreous ganciclovir. Arch. Ophthalmol. 130, 525–527 (2012). 22491927 10.1001/archophthalmol.2011.1615

[35] K. L. Tawse, C. R. Baumal, Intravitreal foscarnet for recurring CMV retinitis in a congenitally infected premature infant. J. AAPOS 18, 78–80 (2014). 24568990 10.1016/j.jaapos.2013.09.015

[36] S. Walker, A. Iguchi, N. P. Jones, Frosted branch angiitis: A review. Eye 18, 527–533 (2004). 15131687 10.1038/sj.eye.6700712

[37] M. Campbell, P. Humphries, The blood-retina barrier: Tight junctions and barrier modulation. Adv. Exp. Med. Biol. 763, 70–84 (2012). 23397619

[38] J. Cunha-Vaz, The blood–retinal barrier in retinal disease. Eur. Ophthal. Rev. 03, 105–108 (2009).

[39] F. O’Leary, M. Campbell, The blood–retina barrier in health and disease. FEBS J. 290, 878–891 (2023). 34923749 10.1111/febs.16330

[40] D. L. Stenkamp, Development of the vertebrate eye and retina. Prog. Mol. Biol. Transl. Sci. 134, 397–414 (2015). 26310167 10.1016/bs.pmbts.2015.06.006 PMC5734922

[41] B. W. Chow, C. Gu, Gradual suppression of transcytosis governs functional blood-retinal barrier formation. Neuron 93, 1325–1333.e3 (2017). 28334606 10.1016/j.neuron.2017.02.043 PMC5480403

[42] R. Daneman, L. Zhou, A. A. Kebede, B. A. Barres, Pericytes are required for blood–brain barrier integrity during embryogenesis. Nature 468, 562–566 (2010). 20944625 10.1038/nature09513 PMC3241506

[43] A. Reichenbach, A. Bringmann, Glia of the human retina. Glia 68, 768–796 (2020). 31793693 10.1002/glia.23727

[44] A. Kumar, R. K. Pandey, L. J. Miller, P. K. Singh, M. Kanwar, Muller glia in retinal innate immunity: A perspective on their roles in endophthalmitis. Crit. Rev. Immunol. 33, 119–135 (2013). 23582059 10.1615/critrevimmunol.2013006618 PMC3697845

[45] A. W. Taylor, Ocular immune privilege. Eye 23, 1885–1889 (2009). 19136922 10.1038/eye.2008.382 PMC4698145

[46] J. J. Hunter, J. I. W. Morgan, W. H. Merigan, D. H. Sliney, J. R. Sparrow, D. R. Williams, The susceptibility of the retina to photochemical damage from visible light. Prog. Retin. Eye Res. 31, 28–42 (2012). 22085795 10.1016/j.preteyeres.2011.11.001 PMC3242847

[47] S. S. Atherton, C. K. Newell, M. Y. Kanter, S. W. Cousins, T cell depletion increases susceptibility to murine cytomegalovirus retinitis. Invest. Ophthalmol. Vis. Sci. 33, 3353–3360 (1992). 1330968

[48] R. D. Dix, E. R. Podack, S. W. Cousins, Loss of the perforin cytotoxic pathway predisposes mice to experimental cytomegalovirus retinitis. J. Virol. 77, 3402–3408 (2003). 12610115 10.1128/JVI.77.6.3402-3408.2003 PMC149521

[49] R. D. Dix, C. O. Ekworomadu, E. Hernandez, S. W. Cousins, Perforin knockout mice, but not mice with MAIDS, show protection against experimental cytomegalovirus retinitis after adoptive transfer of immune cells with a functional perforin cytotoxic pathway. Arch. Virol. 149, 2235–2244 (2004). 15503209 10.1007/s00705-004-0370-3

[50] Y. Lu, J. E. Bigger, C. A. Thomas, S. S. Atherton, Adoptive transfer of murine cytomegalovirus-immune lymph node cells prevents retinitis in T-cell-depleted mice. Invest. Ophthalmol. Vis. Sci. 38, 301–310 (1997). 9040462

[51] J. E. Bigger, M. Tanigawa, C. A. Thomas, S. S. Atherton, Protection against murine cytomegalovirus retinitis by adoptive transfer of virus-specific CD8^+^ T cells. Invest. Ophthalmol. Vis. Sci. 40, 2608–2613 (1999). 10509656

[52] M. Zhang, S. S. Atherton, Apoptosis in the retina during MCMV retinitis in immunosuppressed BALB/c mice. J. Clin. Virol. 25, 137–147 (2002). 10.1016/s1386-6532(02)00102-6 12361764

[53] H. Chien, R. D. Dix, Evidence for multiple cell death pathways during development of experimental cytomegalovirus retinitis in mice with retrovirus-induced immunosuppression: Apoptosis, necroptosis, and pyroptosis. J. Virol. 86, 10961–10978 (2012). 22837196 10.1128/JVI.01275-12 PMC3457157

[54] J. Mo, B. Marshall, J. Covar, N. Y. Zhang, S. B. Smith, S. S. Atherton, M. Zhang, Role of Bax in death of uninfected retinal cells during murine cytomegalovirus retinitis. Invest. Opthalmol. Vis. Sci. 55, 7137–7146 (2014). 10.1167/iovs.14-15404 PMC4224579 25298417

[55] R. D. Dix, C. Cray, S. W. Cousins, Mice immunosuppressed by murine retrovirus infection (MAIDS) are susceptible to cytomegalovirus retinitis. Curr. Eye Res. 13, 587–595 (1994). 7956311 10.3109/02713689408999892

[56] J. J. Carter, J. G. E. Nemeno, J. J. Oh, J. E. Houghton, R. D. Dix, Atypical cytomegalovirus retinal disease in pyroptosis-deficient mice with murine acquired immunodeficiency syndrome. Exp. Eye Res. 209, 108651 (2021). 34097907 10.1016/j.exer.2021.108651 PMC8595509

[57] I. Slavuljica, D. Kveštak, P. Csaba Huszthy, K. Kosmac, W. J. Britt, S. Jonjić, Immunobiology of congenital cytomegalovirus infection of the central nervous system - The murine cytomegalovirus model. Cell. Mol. Immunol. 12, 180–191 (2015). 25042632 10.1038/cmi.2014.51 PMC4654296

[58] J. Xu, X. Liu, X. Zhang, B. Marshall, Z. Dong, S. B. Smith, D. G. Espinosa-Heidmann, M. Zhang, Retinal and choroidal pathologies in aged BALB/c mice following systemic neonatal murine cytomegalovirus infection. Am. J. Pathol. 191, 1787–1804 (2021). 34197777 10.1016/j.ajpath.2021.06.008 PMC8485058

[59] M. Zhang, H. Xin, P. Roon, S. S. Atherton, Infection of retinal neurons during murine cytomegalovirus retinitis. Invest. Opthalmol. Vis. Sci. 46, 2047–2055 (2005). 10.1167/iovs.05-0005 15914622

[60] B. M. Braunger, C. Demmer, E. R. Tamm, Programmed cell death during retinal development of the mouse eye in *Retinal Degenerative Diseases*, Advances in Experimental Medicine and Biology, vol 801 J. Ash, C. Grimm, J. Hollyfield, R. Anderson, M. LaVail, C. Bowes Rickman, C., Eds (Springer, 2014), pp. 9–13. 10.1007/978-1-4614-3209-8_2 24664675

[61] E. Vecino, M. Hernandez, M. Garcia, Cell death in the developing vertebrate retina. Int. J. Dev. Biol. 48, 965–974 (2004). 15558487 10.1387/ijdb.041891ev

[62] Q. Li, B. A. Barres, Microglia and macrophages in brain homeostasis and disease. Nat. Rev. Immunol. 18, 225–242 (2018). 29151590 10.1038/nri.2017.125

[63] M. Colonna, O. Butovsky, Microglia function in the central nervous system during health and neurodegeneration. Annu. Rev. Immunol. 35, 441–468 (2017). 28226226 10.1146/annurev-immunol-051116-052358 PMC8167938

[64] F. Li, D. Jiang, M. A. Samuel, Microglia in the developing retina. Neural Dev. 14, 12 (2019). 31888774 10.1186/s13064-019-0137-x PMC6938006

[65] G. Rathnasamy, W. S. Foulds, E.-A. Ling, C. Kaur, Retinal microglia – A key player in healthy and diseased retina. Prog. Neurobiol. 173, 18–40 (2019). 29864456 10.1016/j.pneurobio.2018.05.006

[66] E. Mass, F. Nimmerjahn, K. Kierdorf, A. Schlitzer, Tissue-specific macrophages: How they develop and choreograph tissue biology. Nat. Rev. Immunol. 23, 563–579 (2023). 36922638 10.1038/s41577-023-00848-y PMC10017071

[67] F. Giovannoni, F. J. Quintana, The role of astrocytes in CNS inflammation. Trends Immunol. 41, 805–819 (2020). 32800705 10.1016/j.it.2020.07.007 PMC8284746

[68] M. E. Lull, M. L. Block, Microglial activation and chronic neurodegeneration. Neurotherapeutics 7, 354–365 (2010). 20880500 10.1016/j.nurt.2010.05.014 PMC2951017

[69] S. C. Woodburn, J. L. Bollinger, E. S. Wohleb, The semantics of microglia activation: Neuroinflammation, homeostasis, and stress. J. Neuroinflammation 18, 258 (2021). 34742308 10.1186/s12974-021-02309-6 PMC8571840

[70] T. F. Ng, J. W. Streilein, Light-induced migration of retinal microglia into the subretinal space. Invest. Ophthalmol. Vis. Sci. 42, 3301–3310 (2001). 11726637

[71] W. S. Carbonell, S.-I. Murase, A. F. Horwitz, J. W. Mandell, Migration of perilesional microglia after focal brain injury and modulation by CC chemokine receptor 5: An in situ time-lapse confocal imaging study. J. Neurosci. 25, 7040–7047 (2005). 16049180 10.1523/JNEUROSCI.5171-04.2005 PMC6724831

[72] F. Yu, Y. Wang, A. R. Stetler, R. K. Leak, X. Hu, J. Chen, Phagocytic microglia and macrophages in brain injury and repair. CNS Neurosci. Ther. 28, 1279–1293 (2022). 35751629 10.1111/cns.13899 PMC9344092

[73] B. A. Bell, C. Kaul, V. L. Bonilha, M. E. Rayborn, K. Shadrach, J. G. Hollyfield, The BALB/c mouse: Effect of standard vivarium lighting on retinal pathology during aging. Exp. Eye Res. 135, 192–205 (2015). 25895728 10.1016/j.exer.2015.04.009 PMC4446204

[74] J. L. Ridet, A. Privat, S. K. Malhotra, F. H. Gage, Reactive astrocytes: Cellular and molecular cues to biological function. Trends Neurosci. 20, 570–577 (1997). 9416670 10.1016/s0166-2236(97)01139-9

[75] M. A. Dyer, C. L. Cepko, Control of Müller glial cell proliferation and activation following retinal injury. Nat. Neurosci. 3, 873–880 (2000). 10966617 10.1038/78774

[76] R. E. MacLaren, Development and role of retinal glia in regeneration of ganglion cells following retinal injury. Br. J. Ophthalmol. 80, 458–464 (1996). 8695569 10.1136/bjo.80.5.458 PMC505499

[77] S. A. Liddelow, K. A. Guttenplan, L. E. Clarke, F. C. Bennett, C. J. Bohlen, L. Schirmer, M. L. Bennett, A. E. Münch, W. S. Chung, T. C. Peterson, D. K. Wilton, A. Frouin, B. A. Napier, N. Panicker, M. Kumar, M. S. Buckwalter, D. H. Rowitch, V. L. Dawson, T. M. Dawson, B. Stevens, B. A. Barres, Neurotoxic reactive astrocytes are induced by activated microglia. Nature 541, 481–487 (2017). 28099414 10.1038/nature21029 PMC5404890

[78] K. A. Guttenplan, B. K. Stafford, R. N. El-Danaf, D. I. Adler, A. E. Münch, M. K. Weigel, A. D. Huberman, S. A. Liddelow, Neurotoxic reactive astrocytes drive neuronal death after retinal injury. Cell Rep. 31, 107776 (2020). 32579912 10.1016/j.celrep.2020.107776 PMC8091906

[79] M. L. Block, L. Zecca, J.-S. Hong, Microglia-mediated neurotoxicity: Uncovering the molecular mechanisms. Nat. Rev. Neurosci. 8, 57–69 (2007). 17180163 10.1038/nrn2038

[80] O. Kann, F. Almouhanna, B. Chausse, Interferon γ: A master cytokine in microglia-mediated neural network dysfunction and neurodegeneration. Trends Neurosci. 45, 913–927 (2022). 36283867 10.1016/j.tins.2022.10.007

[81] I. E. Papageorgiou, A. Lewen, L. V. Galow, T. Cesetti, J. Scheffel, T. Regen, U. K. Hanisch, O. Kann, TLR4-activated microglia require IFN-γ to induce severe neuronal dysfunction and death in situ. Proc. Natl. Acad. Sci. U.S.A. 113, 212–217 (2016). 26699475 10.1073/pnas.1513853113 PMC4711883

[82] J. Booss, P. R. Dann, B. P. Griffith, J. H. Kim, Glial nodule encephalitis in the guinea pig: Serial observations following cytomegalovirus infection. Acta Neuropathol. 75, 465–473 (1988). 2837038 10.1007/BF00687133

[83] J. Booss, S. R. Winkler, B. P. Griffith, J. H. Kim, Viremia and glial nodule encephalitis after experimental systemic cytomegalovirus infection. Lab. Invest. 61, 644–649 (1989). 2557488

[84] J. Booss, P. R. Dann, S. R. Winkler, B. P. Griffith, J. H. Kim, Mechanisms of injury to the central nervous system following experimental cytomegalovirus infection. Am. J. Otolaryngol. 11, 313–317 (1990). 2176065 10.1016/0196-0709(90)90061-y

[85] C. Fernández-Alarcón, L. E. Meyer, M. A. McVoy, J. R. Lokensgard, S. Hu, M. A. Benneyworth, K. M. Anderholm, B. C. Janus, M. R. Schleiss, Impairment in neurocognitive function following experimental neonatal guinea pig cytomegalovirus infection. Pediatr. Res. 89, 838–845 (2021). 32555536 10.1038/s41390-020-1010-7 PMC8168912

[86] H. Kawasaki, I. Kosugi, M. Sakao-Suzuki, S. Meguro, Y. Arai, Y. Tsutsui, T. Iwashita, Cytomegalovirus initiates infection selectively from high-level β1 integrin–expressing cells in the brain. Am. J. Pathol. 185, 1304–1323 (2015). 25797647 10.1016/j.ajpath.2015.01.032

[87] M. Nobuloni, A. Pellegrinelli, A. Ferri, A. Tosoni, S. Bonetto, P. Zorbi, R. Boldorini, L. Vago, G. Costanzi, Etiology of microglial nodules in brains of patients with acquired immunodeficiency syndrome. J. Neurovirol. 6, 46–50 (2000). 10786996 10.3109/13550280009006381

[88] J. D. Cherry, G. Meng, S. Daley, W. Xia, S. Svirsky, V. E. Alvarez, R. Nicks, M. Pothast, H. Kelley, B. Huber, Y. Tripodis, M. L. Alosco, J. Mez, A. C. McKee, T. D. Stein, CCL2 is associated with microglia and macrophage recruitment in chronic traumatic encephalopathy. J. Neuroinflammation 17, 370 (2020). 33278887 10.1186/s12974-020-02036-4 PMC7718711

[89] M. He, H. Dong, Y. Huang, S. Lu, S. Zhang, Y. Qian, W. Jin, Astrocyte-derived CCL2 is associated with M1 activation and recruitment of cultured microglial cells. Cell. Physiol. Biochem. 38, 859–870 (2016). 26910882 10.1159/000443040

[90] M. C.-J. Cheeran, S. Hu, S. L. Yager, G. Gekker, P. K. Peterson, J. R. Lokensgard, Cytomegalovirus induces cytokine and chemokine production differentially in microglia and astrocytes: Antiviral implications. J. Neurovirol. 7, 135–147 (2001). 11517386 10.1080/13550280152058799

[91] A. Strack, V. Asensio, I. Campbell, D. Schlüter, M. Deckert, Chemokines are differentially expressed by astrocytes, microglia and inflammatory leukocytes in toxoplasma encephalitis and critically regulated by interferon-γ. Acta Neuropathol. 103, 458–468 (2002). 11935261 10.1007/s00401-001-0491-7

[92] B. Capuccini, J. Lin, C. Talavera-López, S. M. Khan, J. Sodenkamp, R. Spaccapelo, J. Langhorne, Transcriptomic profiling of microglia reveals signatures of cell activation and immune response, during experimental cerebral malaria. Sci. Rep. 6, 39258 (2016). 27991544 10.1038/srep39258 PMC5171943

[93] C. G. Fresta, A. Fidilio, G. Caruso, F. Caraci, F. J. Giblin, G. Marco Leggio, S. Salomone, F. Drago, C. Bucolo, A new human blood–retinal barrier model based on endothelial cells, pericytes, and astrocytes. Int. J. Mol. Sci. 21, 1636 (2020). 32121029 10.3390/ijms21051636 PMC7084779

[94] N. J. Abbott, L. Rönnbäck, E. Hansson, Astrocyte–endothelial interactions at the blood–brain barrier. Nat. Rev. Neurosci. 7, 41–53 (2006). 16371949 10.1038/nrn1824

[95] M. Munro, T. Yadavalli, C. Fonteh, S. Arfeen, A.-M. Lobo-Chan, Cytomegalovirus retinitis in HIV and non-HIV individuals. Microorganisms 8, 55 (2019). 31905656 10.3390/microorganisms8010055 PMC7022607

[96] V. Venturi, K. Nzingha, T. G. Amos, W. C. Charles, I. Dekhtiarenko, L. Cicin-Sain, M. P. Davenport, B. D. Rudd, The neonatal CD8^+^ T cell repertoire rapidly diversifies during persistent viral infection. J. Immunol. 196, 1604–1616 (2016). 26764033 10.4049/jimmunol.1501867 PMC4744528

[97] L. Gibson, C. M. Barysauskas, M. McManus, S. Dooley, D. Lilleri, D. Fisher, T. Srivastava, D. J. Diamond, K. Luzuriaga, Reduced frequencies of polyfunctional CMV-specific T cell responses in infants with congenital CMV infection. J. Clin. Immunol. 35, 289–301 (2015). 25712611 10.1007/s10875-015-0139-3 PMC4366322

[98] M. G. Capretti, C. Marsico, A. Chiereghin, L. Gabrielli, A. Aceti, T. Lazzarotto, Immune monitoring using QuantiFERON®-CMV assay in congenital cytomegalovirus infection: Correlation with clinical presentation and CMV DNA load. Clin. Infect. Dis. 73, 367–373 (2021). 32504086 10.1093/cid/ciaa704

[99] M. Soriano-Ramos, R. Pedrero-Tomé, E. Giménez-Quiles, E. Albert, F. Baquero-Artigao, P. Rodríguez-Molino, T. del Rosal, A. Noguera-Julian, C. Fortuny, M. Ríos-Barnés, J. Saavedra-Lozano, E. Dueñas, M. Sánchez-Mateos, L. Castells, M. de la Serna, M. A. Frick, J. de Vergas, N. Núñez-Enamorado, M. T. Moral-Pumarega, M. D. Folgueira, D. Navarro, D. Blázquez-Gamero, CYTRIC Study Group, T-cell immune responses in newborns and long-term sequelae in congenital cytomegalovirus infection (CYTRIC study). J. Pediatr. 272, 114084 (2024). 38705230 10.1016/j.jpeds.2024.114084

[100] M. Karlstetter, R. Scholz, M. Rutar, W. T. Wong, J. M. Provis, T. Langmann, Retinal microglia: Just bystander or target for therapy? Prog. Retin. Eye Res. 45, 30–57 (2015). 25476242 10.1016/j.preteyeres.2014.11.004

[101] J. Silver, J. H. Miller, Regeneration beyond the glial scar. Nat. Rev. Neurosci. 5, 146–156 (2004). 14735117 10.1038/nrn1326

[102] J. R. Faulkner, J. E. Herrmann, M. J. Woo, K. E. Tansey, N. B. Doan, M. V. Sofroniew, Reactive astrocytes protect tissue and preserve function after spinal cord injury. J. Neurosci. 24, 2143–2155 (2004). 14999065 10.1523/JNEUROSCI.3547-03.2004 PMC6730429

[103] C. L. Anthony, J. C. Bavinger, S. Yeh, Advances in the diagnosis and management of acute retinal necrosis. Ann. Eye Sci. 5, 28–28 (2020). 33381683 10.21037/aes-2019-dmu-09 PMC7771653

[104] G. L. Darmstadt, E. M. Keithley, J. P. Harris, Effects of cyclophosphamide on the pathogenesis of cytomegalovirus-induced labyrinthitis. Ann. Otol. Rhinol. Laryngol. 99, 960–968 (1990). 2173893 10.1177/000348949009901206

[105] K. Guo, D. J. K. Yombo, Z. Wang, Z. Navaeiseddighi, J. Xu, T. Schmit, N. Ahamad, J. Tripathi, B. De Kumar, R. Mathur, J. Hur, J. Sun, M. A. Olszewski, N. Khan, The chemokine receptor CXCR3 promotes CD8^+^ T cell–dependent lung pathology during influenza pathogenesis. Sci. Adv. 10, eadj1120 (2024). 38170765 10.1126/sciadv.adj1120 PMC10776024

[106] K. A. Zurbach, T. Moghbeli, C. M. Snyder, Resolving the titer of murine cytomegalovirus by plaque assay using the M2-10B4 cell line and a low viscosity overlay. Virol. J. 11, 71 (2014). 24742045 10.1186/1743-422X-11-71 PMC4006460

[107] C. M. Snyder, J. E. Allan, E. L. Bonnett, C. M. Doom, A. B. Hill, Cross-presentation of a spread-defective mcmv is sufficient to prime the majority of virus-specific CD8^+^ T Cells. PLOS ONE 5, e9681 (2010). 20300633 10.1371/journal.pone.0009681 PMC2837378

[108] M. J. Reddehase, M. R. Fibi, G. M. Keil, U. H. Koszinowski, Late-phase expression of a murine cytomegalovirus immediate-early antigen recognized by cytolytic T lymphocytes. J. Virol. 60, 1125–1129 (1986). 2431160 10.1128/jvi.60.3.1125-1129.1986 PMC253362

[109] H. Morrison, K. Young, M. Qureshi, R. K. Rowe, J. Lifshitz, Quantitative microglia analyses reveal diverse morphologic responses in the rat cortex after diffuse brain injury. Sci. Rep. 7, 13211 (2017). 29038483 10.1038/s41598-017-13581-z PMC5643511

[110] B. Abu-Jamous, S. Kelly, Clust: Automatic extraction of optimal co-expressed gene clusters from gene expression data. Genome Biol. 19, 172 (2018). 30359297 10.1186/s13059-018-1536-8 PMC6203272

[111] S. Hänzelmann, R. Castelo, J. Guinney, GSVA: Gene set variation analysis for microarray and RNA-Seq data. BMC Bioinformatics 14, 7 (2013). 23323831 10.1186/1471-2105-14-7 PMC3618321

