# Supplementary material for: Immune responses drive chorioretinitis and retinal pathology after neonatal CMV infection
Source: Sci Adv. 2024 Nov 20;10(47):eadn6379. doi: 10.1126/sciadv.adn6379 (PMC11578184; doi:10.1126/sciadv.adn6379)
Supplement: Supplementary file 1 — Figs. S1 to S12 Tables S1 to S4 Legend for data file S1 Legend for movie S1 [file sciadv.adn6379_sm.pdf]

Science Advances
AAAS

Supplementary Materials for

Immune responses drive chorioretinitis and retinal pathology after neonatal CMV infection

Jessica L. McCord *et al.*

Corresponding author: Christopher M. Snyder, christopher.snyder@jefferson.edu

Sci. Adv. **10**, eadn6379 (2024)
DOI: 10.1126/sciadv.adn6379

The PDF file includes:

Figs. S1 to S12
Tables S1 to S4
Legend for movie S1
Legend for data file S1

Other Supplementary Material for this manuscript includes the following:

Movie S1
Data file S1

Supplemental Figures

Fig. S1.

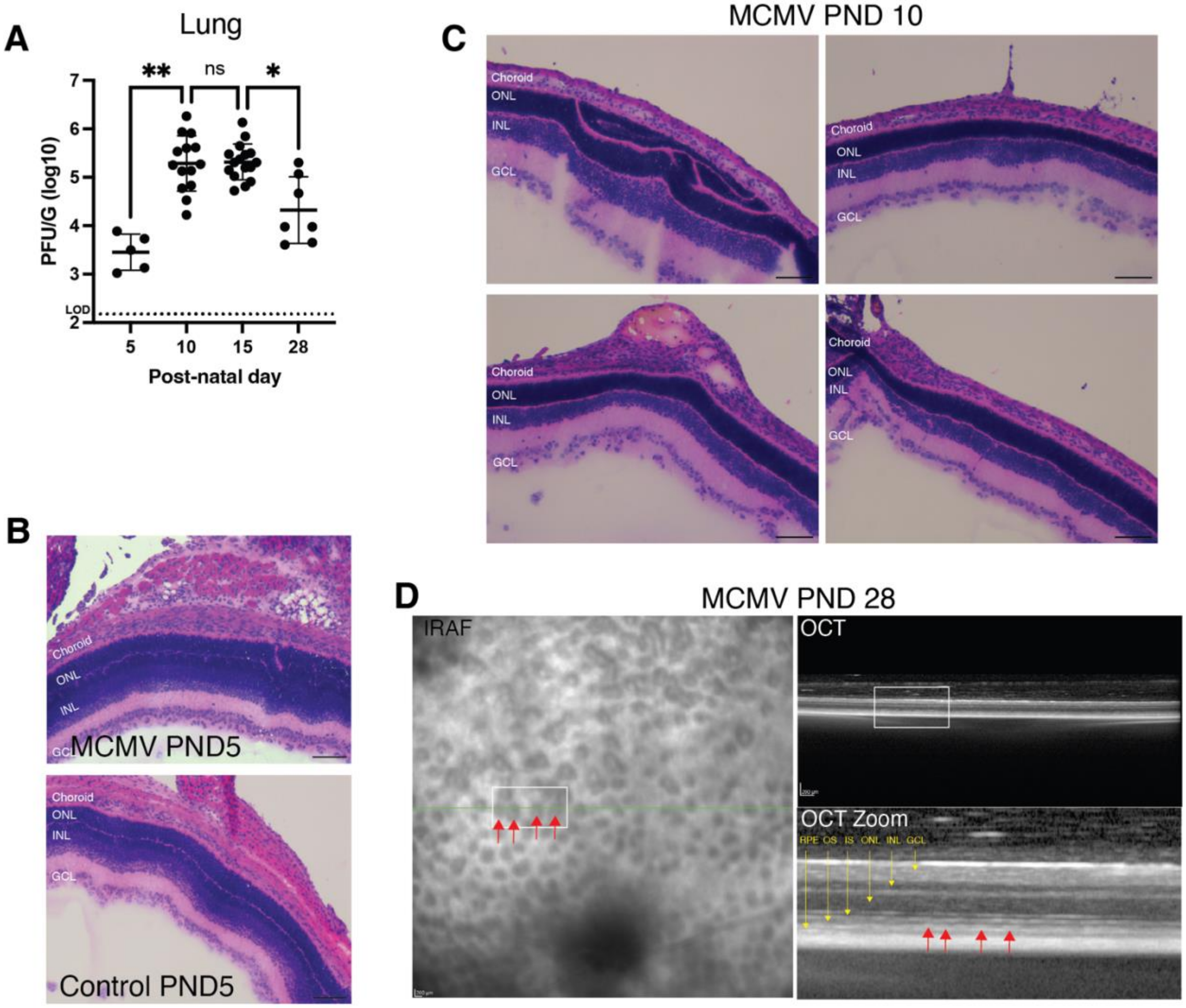

Fig S1: (A) MCMV titer in lung tissue of mice infected with MCMV over time measured by plaque assay and presented as PFU/g lung tissue (LOD = 150 plaques/g). Analyzed by one-way ANOVA with multiple comparisons *p < 0.05 **p <0.01 ***p < 0.001 ****p < 0.0001. n=5-13 mice per timepoint. **(B)** H&E staining of eyes from mice infected with MCMV or PBS control at PND5 (representative of n=5). **(C)** H&E staining of serial sections from one eye of one MCMV-infected mouse, demonstrating focal pathology at PND10. **(D)** SLO image of MCMV infected mouse at PND28. OCT image was taken at the green line on SLO. White box highlighting the same area as OCT zoom on all images. Red arrows denote photoreceptor infoldings. Representative of n=3 mice. Scale bars indicate 100 µm.

Fig. S2.

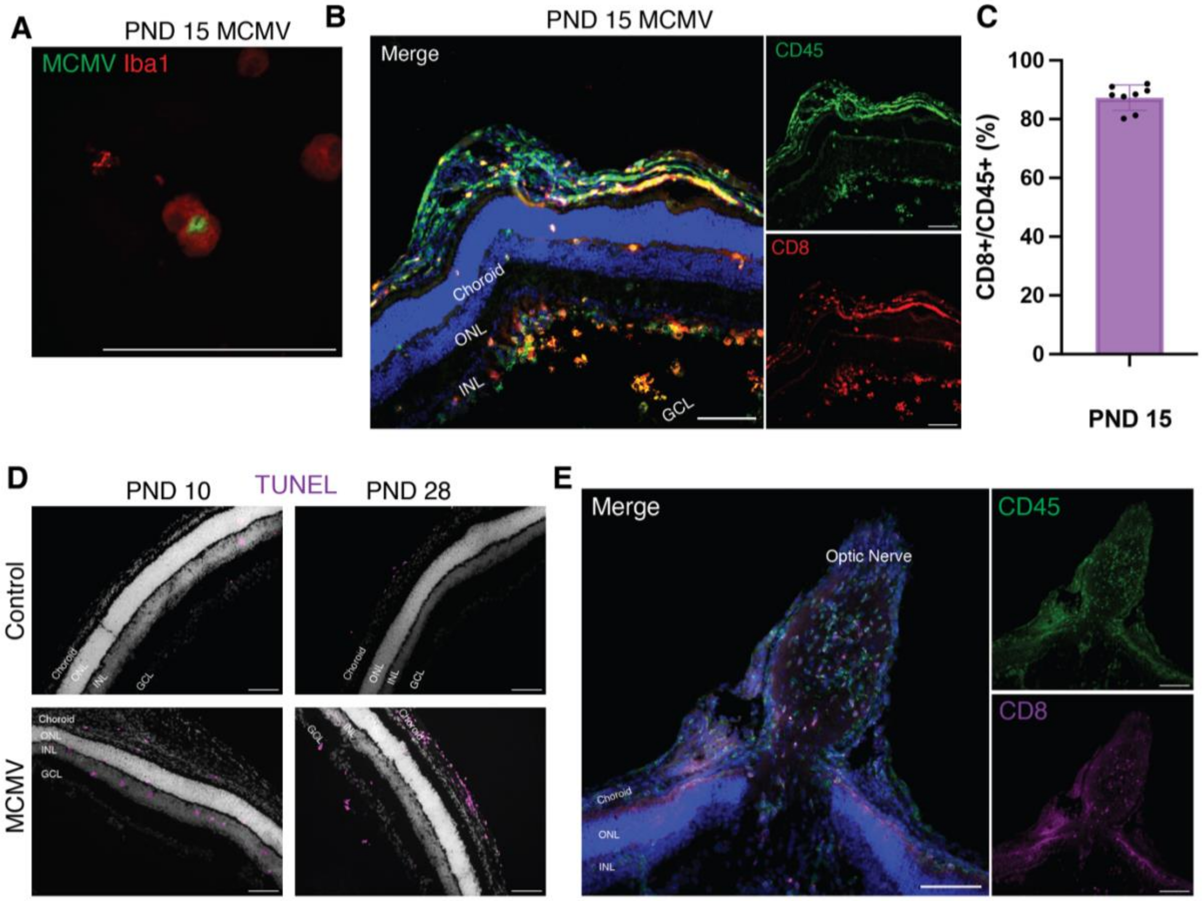

Fig S2: (A) Representative immunofluorescent staining of retinal flatmount of Iba1^{+} MCMV^{+} (pp89^{+}) cell. **(B)** Representative immunofluorescent staining of thin sections showing CD45^{+} and CD8^{+} cells in an MCMV infected mouse at PND15. **(C)** Percent of CD45^{+} cells that are CD8^{+} calculated from retinal flatmounts, n= 8 mice. **(D)** Representative TUNEL staining at PND10 and PND28. **(E)** Representative immunofluorescent staining of thin sections showing CD45^{+} and CD8^{+} cells in the optic nerve in an MCMV infection mouse at PND15. Scale bars indicate 100µm. Images are representative of n=3-8 mice.

Fig. S3.

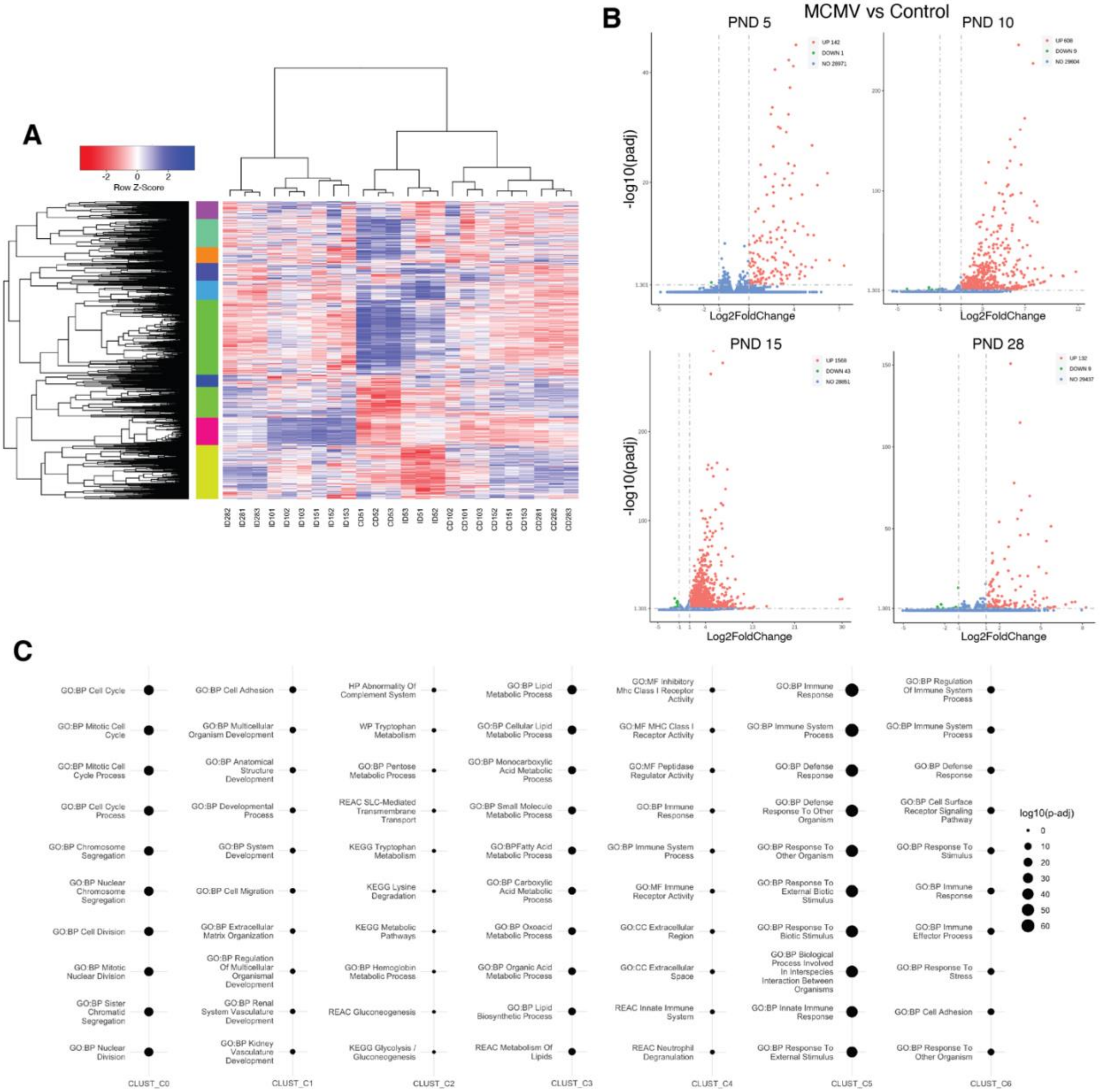

Fig S3: (A) Heatmap of Z-scores determined by row gene expression of all differentially expressed genes across all time points. Samples named with C = control uninfected mice. Samples named with I = infected mice. D = Day. The first two digits of sample name indicate the post-natal day the sample was collected. The last digit is the mouse number. Therefore ID282 = Infected, Day 28, mouse 2. **(B)** Individual volcano plots of all genes from MCMV-infected eyes vs PBS-treated control eyes at each time point. Plotted based on log2 fold change and log10 of the adjusted p value. Red and green dots indicate significant positive and negative expression (significance = >.05 p value and > ±1 log_2 fold change). **(C)** Top 10 go enrichment pathways on each cluster from co-expression cluster analysis (**Fig 3A**). Dot size denotes significant enrichment (log10 of adjusted p value).

Fig. S4.

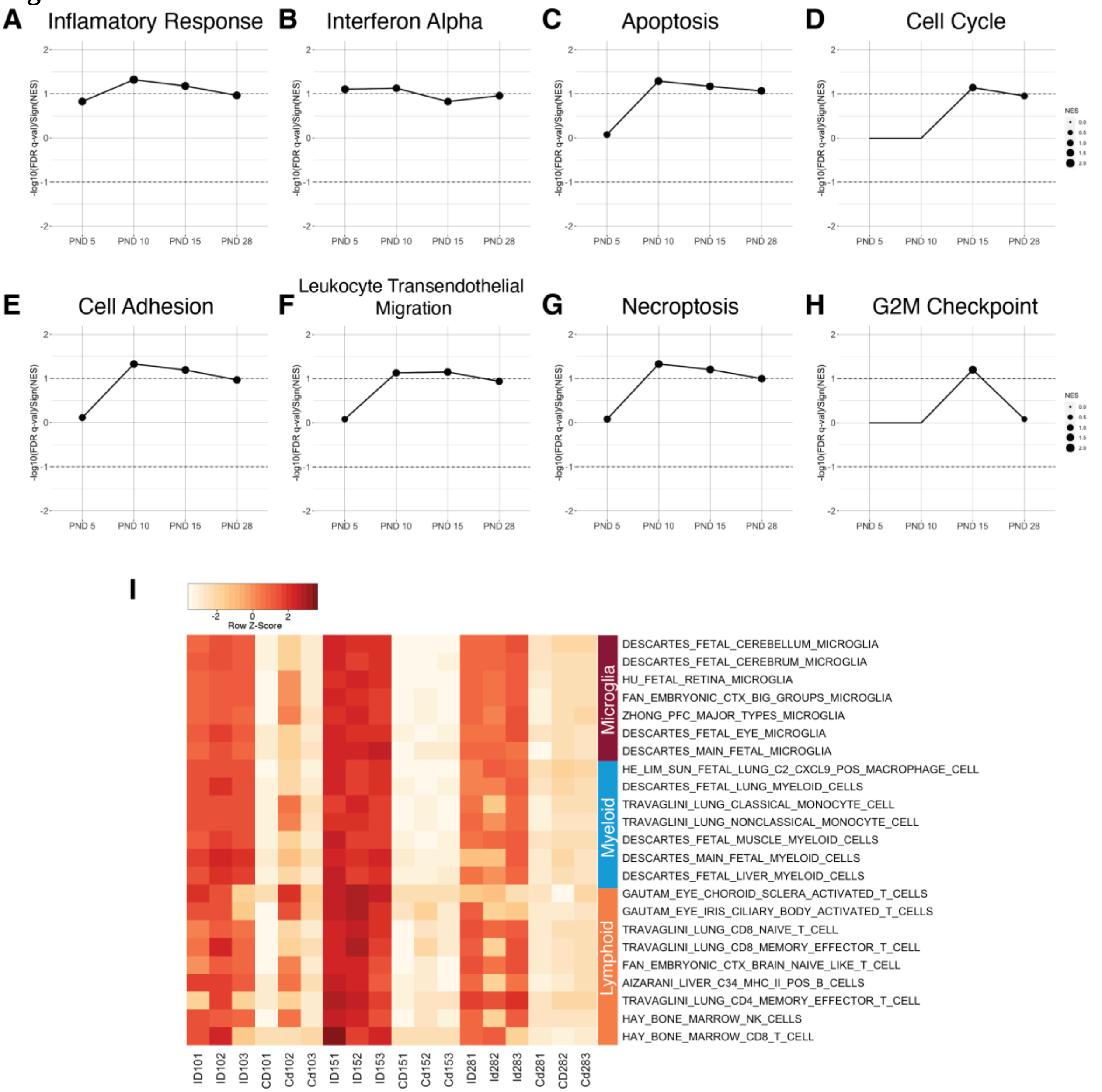

Fig S4: (A to H) Differential expression analysis data from eyes at PND 5, PND 10, PND 15, and PND 28 analyzed by GSEA. Directional significance of enrichment was plotted over time. **(A to D)** Enrichment for Hallmark genes (MSibDB H: Hallmark gene set) **(E to H)** Enrichment for KEGG pathways (MSibDB CP: KEGG). Dotted lines represent calculated statistics for FDR q-val = 0.1 for enrichment; any point outside of dotted lines has an FDR q-val < 0.1. Size of dot at each time point denotes NES. **(I)** Heatmap of GSVA enrichment scores for cell signature gene sets (MSibDB C8: cell type signature gene sets) across all time points. Z-score based on row values. Sample names as in **Fig S3**.

Fig. S5.

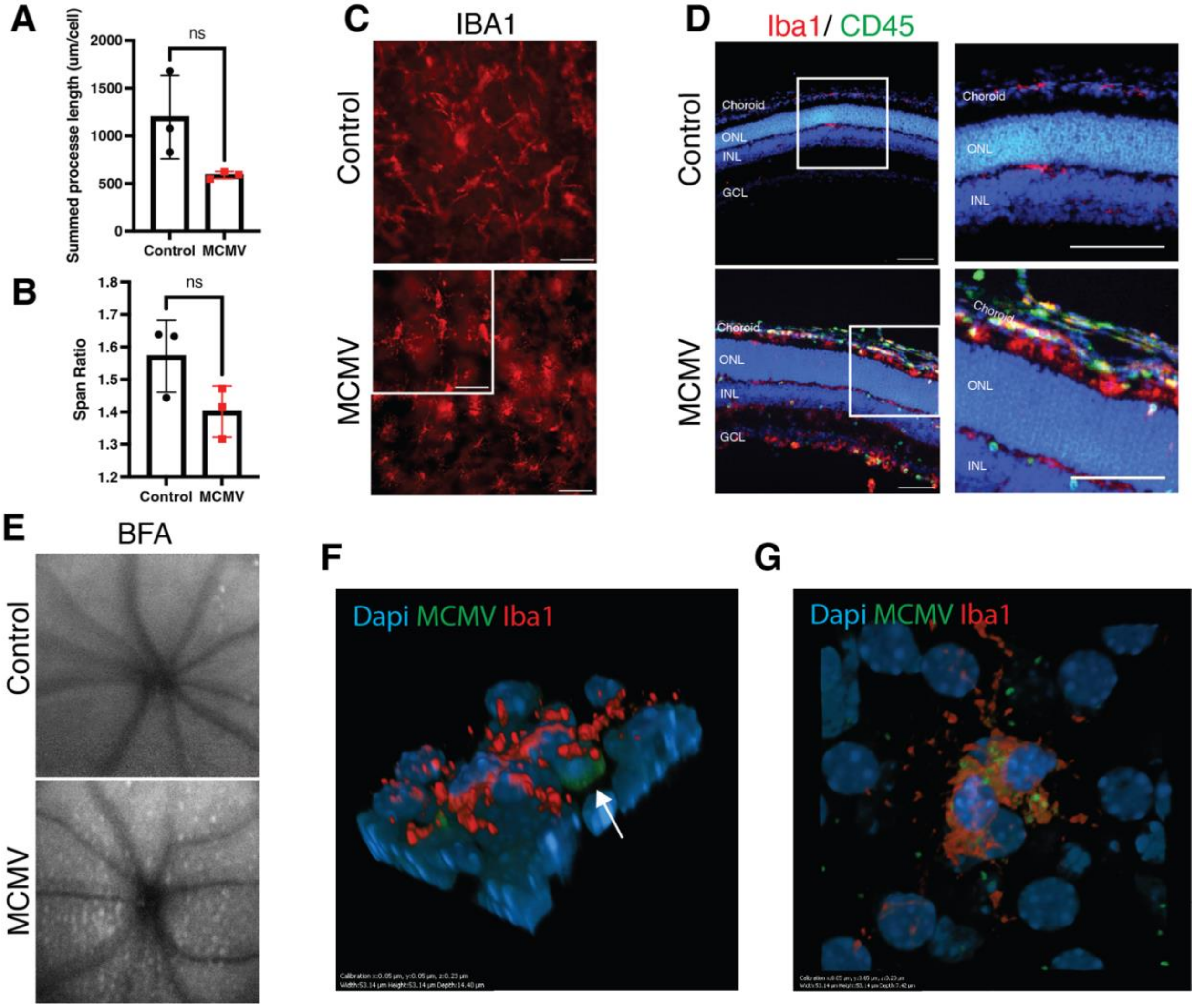

Fig S5: (A&B) Morphological analysis of Iba1^{+} microglia from MCMV and control retinas as assessed by: **(A)** Summed processes length (µm/cell), and **(B)** Span Ratio. Each data point is an average of n>15 images. n=3 mice per group. Analyzed by unpaired t-test; $*p < 0.05$ $**p < 0.01$ $***p < 0.001$ $****p < 0.0001$.**(C)** RPE flat mounts from MCMV infected or control mice stained for Iba1^{+} cells. Magnified area showing microglia in subretinal space (Scale bars = 50 µm). **(D)** Thin sections from MCMV infected and control eyes stained for Iba1^{+} and CD45^{+}. White box denotes magnified area shown to the right. Scale bars indicate 100 µm **(E)** Blue Autofluorescence images of MCMV and control eyes at PND28. **(F-G)** Representative confocal 3D projected image of RPE flatmounts stained with Iba1 and MCMV (pp89) **(F)** Shows a microglia (Iba1^{+}) cell on the inner RPE surface, white arrow denotes RPE cell infected with MCMV. **(G)** Shows two microglia cells on the inner RPE surface with MCMV positive staining in cytoplasm.

Fig. S6.

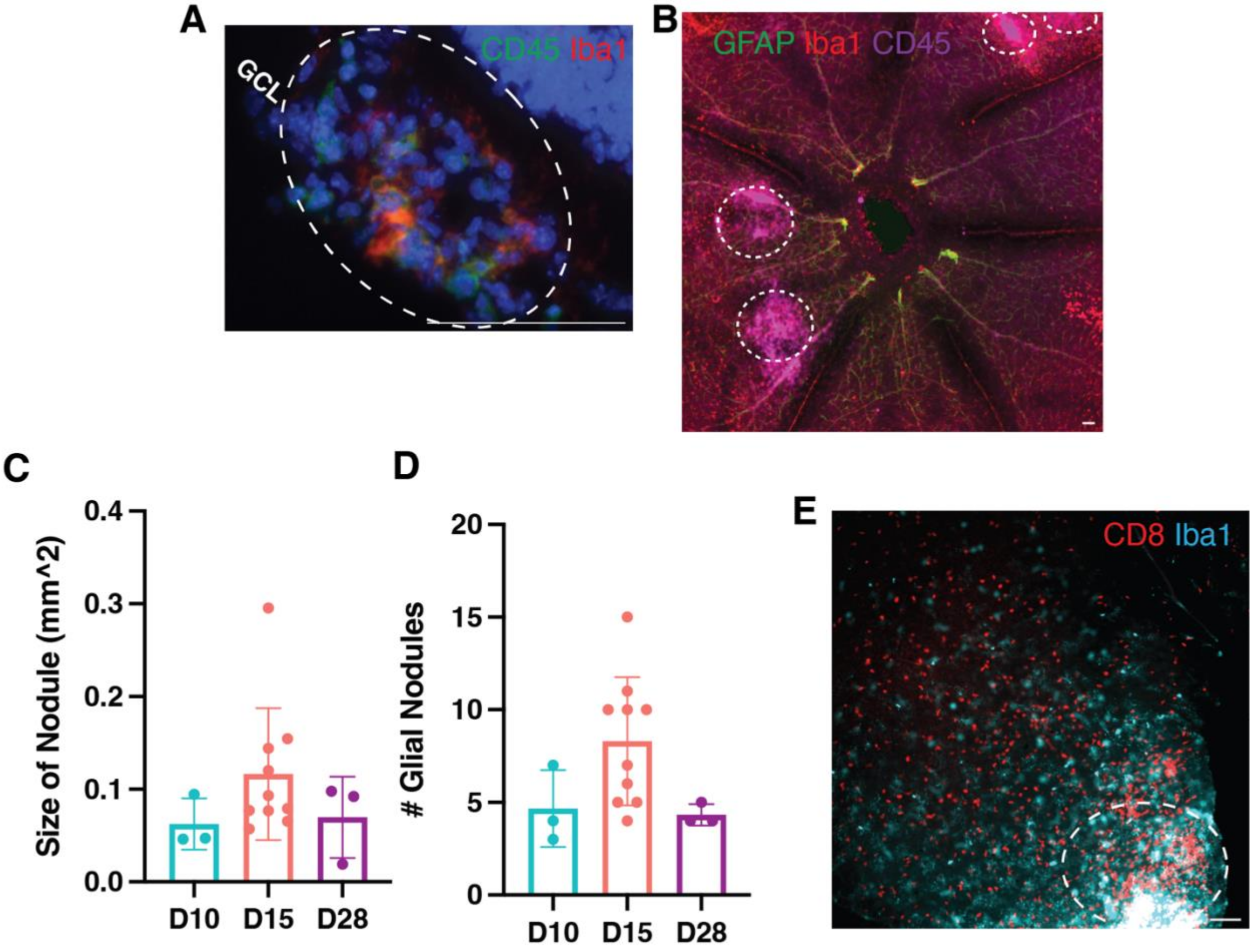

Fig S6: (A) Glial nodule from an MCMV-infected mouse at PND15, stained for $CD45^{+}$ and $Iba1^{+}$ cells. White circle denotes nodule. Representative of n=5 mice. **(B)** Retinal flat mount from an MCMV-infected mouse at PND15 stained with antibodies specific for GFAP, Iba1, and CD45. White circles outline glial nodules. Representative of n=5 mice. **(C)** Average size of glial nodules at each time point as measured on retinal flat mounts from MCMV-infected mice. Each data point represents the average size of nodules from a single mouse at the indicated timepoint n=3-10 mice per group. **(D)** Number of glial nodules per eye at each time point. **(E)** Retinal flat mount of an eye from an MCMV infected mouse at PND15 stained for $CD8^{+}$ and $Iba1^{+}$ cells and used to make the contour plot in **Fig 5F**. White circle denotes the nodule. Representative of n=3 mice. Scale bars throughout = 100 μm.

Fig. S7.

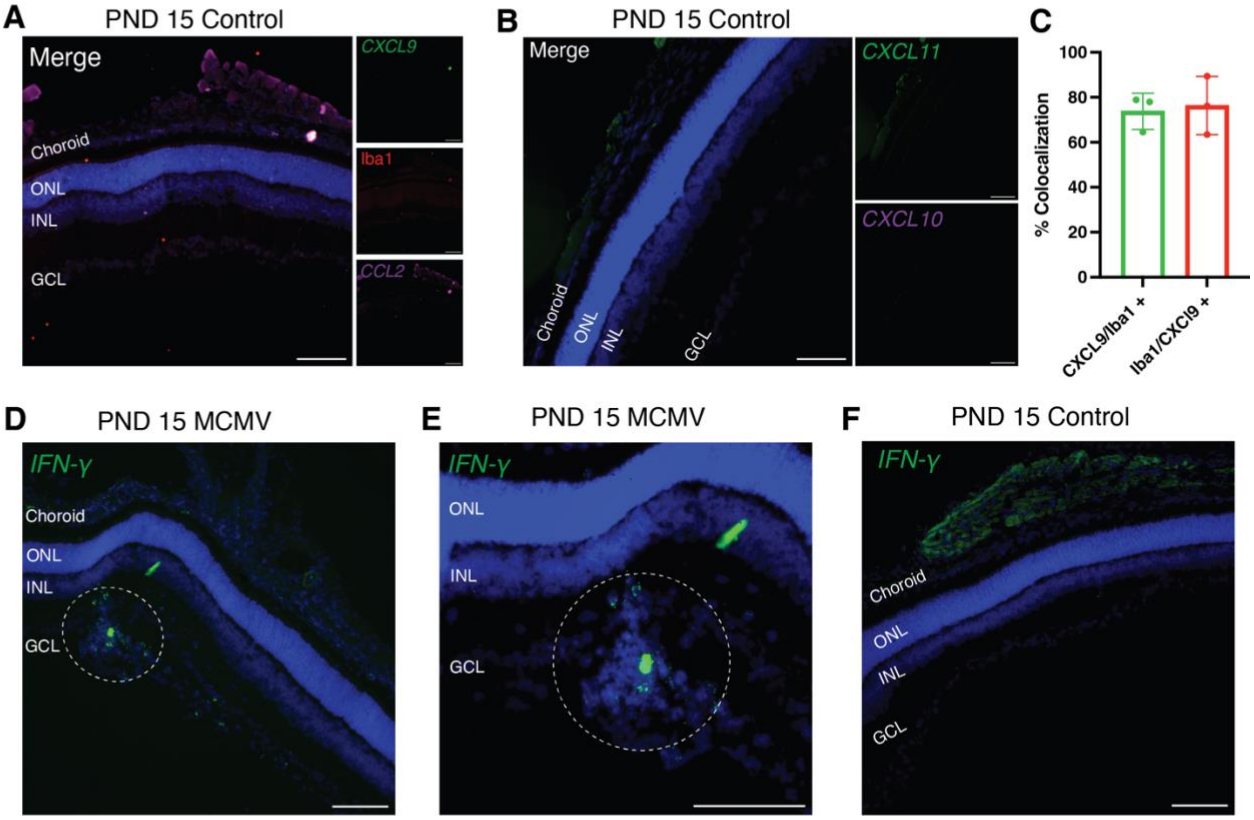

Fig S7: (A & B) In situ hybridization (RNAScope) of mRNA expression in control (uninfected) eyes. **(A)** Sections were probed for *CXCL9* and *CCL2* transcripts and co-stained $Iba1^+$ cells. **(B)** Sections were probed for *CXCL11* and *CXCL10* transcripts. **(C)** Colocalization of *CXCL9* transcripts and Iba1 staining was measured as the percent of *CXCL9* that colocalized with Iba1 (CXCL9/Iba1+) and as the inverse, the percent of $Iba1^+$ cells that colocalized with CXCL9 (Iba1/CXCL9+). Each data point represents an average co-localization in a section from n=3 mice per group. **(D-F)** In situ hybridization (RNAScope) for *IFN-γ* transcripts in eyes from MCMV infected or uninfected control mice. Data are representative of n=3 mice per group. Shown are: **(D)** An eye from an MCMV infected mouse at PND15. White circle denotes glial nodule. **(E)** Magnified image of the glial nodule from **(D). (F)** An eye from an uninfected control mouse at PND15.

Fig. S8.

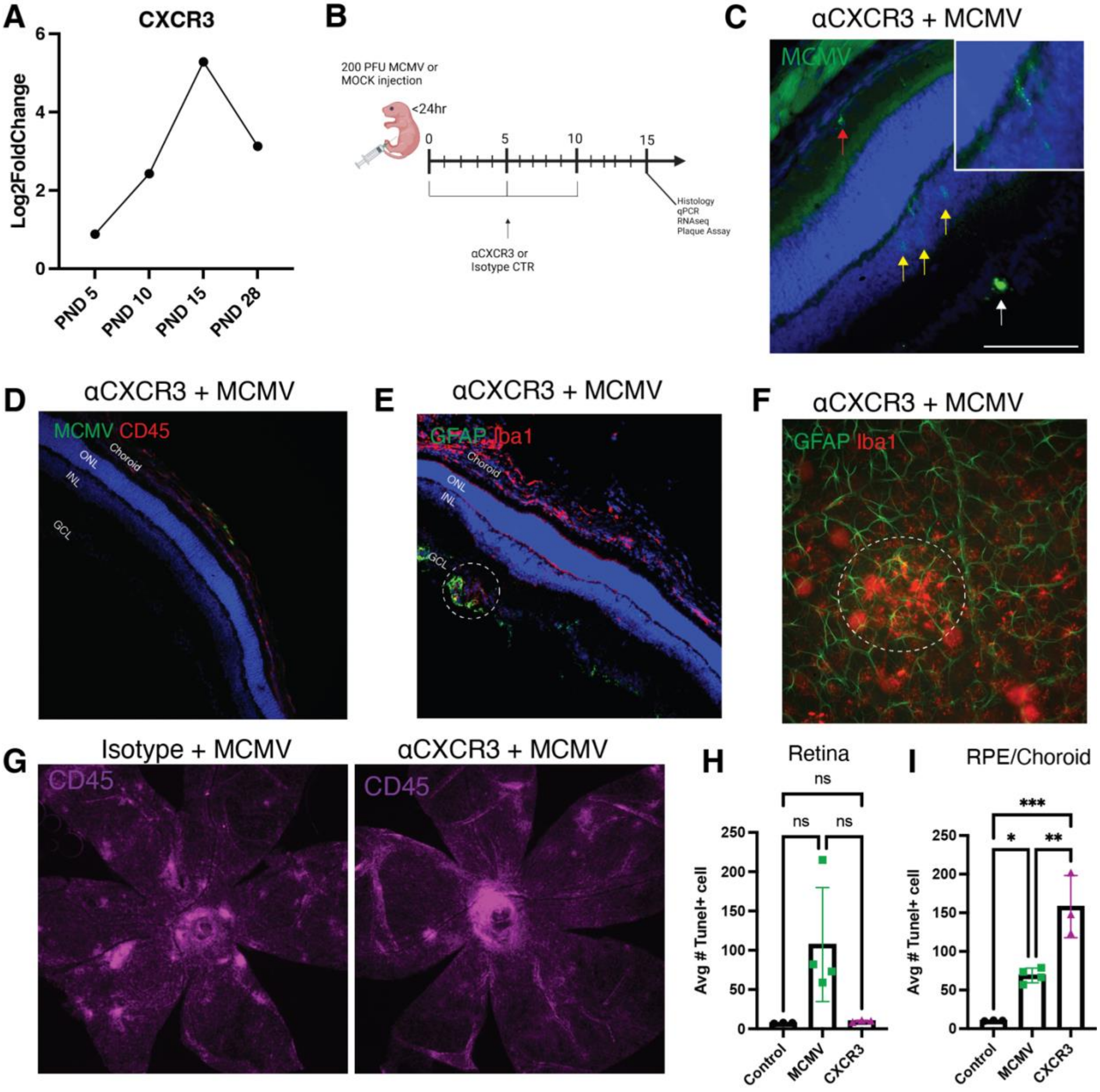

Fig S8: (A) CXCR3 expression over time was assessed by RNA-Seq (as in **Fig 3**). Data show the differential gene expression (Log2 fold change) from infected eyes compared to controls, n=3 samples per group. **(B)** Schematic of infection and antibody treatment timing for CXCR3 blocking experiments. **(C)** Thin section stained for MCMV (pp89). Arrows denote positive staining (white arrow: infected cell in the ganglion cell layer; yellow arrow: punctate pp89 staining within a cell transversing neuroretina, consistent with phagocytosed material within Müller glia; red arrow: punctate pp89 staining within a cell in photoreceptor area, consistent with phagocytosed material within a microglia on the inner face of the RPE) **(D-F)** Representative data from mice infected with MCMV on PND15 and treated with αCXCR3 antibody showing: **(D)** a thin section stained for MCMV (pp89) and CD45, **(E)** a thin section stained for GFAP and Iba1 (white dotted circle denotes a nodule) or **(F)** a flatmount stained for GFAP and Iba1 (white dotted circle, nodule). **(G)** Flat mounts from MCMV-infected mice on PND15 treated with

isotype control antibody (left) or αCXCR3 antibody (right) and stained for CD45. Scale bars for **C-F** indicate 100μm. **(H & I)** Quantification of TUNEL staining of eyes from control (uninfected) mice, MCMV-infected mice or MCMV-infected mice treated with αCXCR3 antibodies. Each data point represents an average number of $TUNEL^+$ cells per image as calculated from 9 images per mouse and n=2-4 mice per condition Significance was analyzed by unpaired t-test; *p < 0.05 **p <0.01 ***p < 0.001 ****p < 0.0001. Data shows: **(H)** $TUNEL^+$ cells in the retina. **(I)** $TUNEL^+$ cells in the RPE/choroid.

Fig. S9.

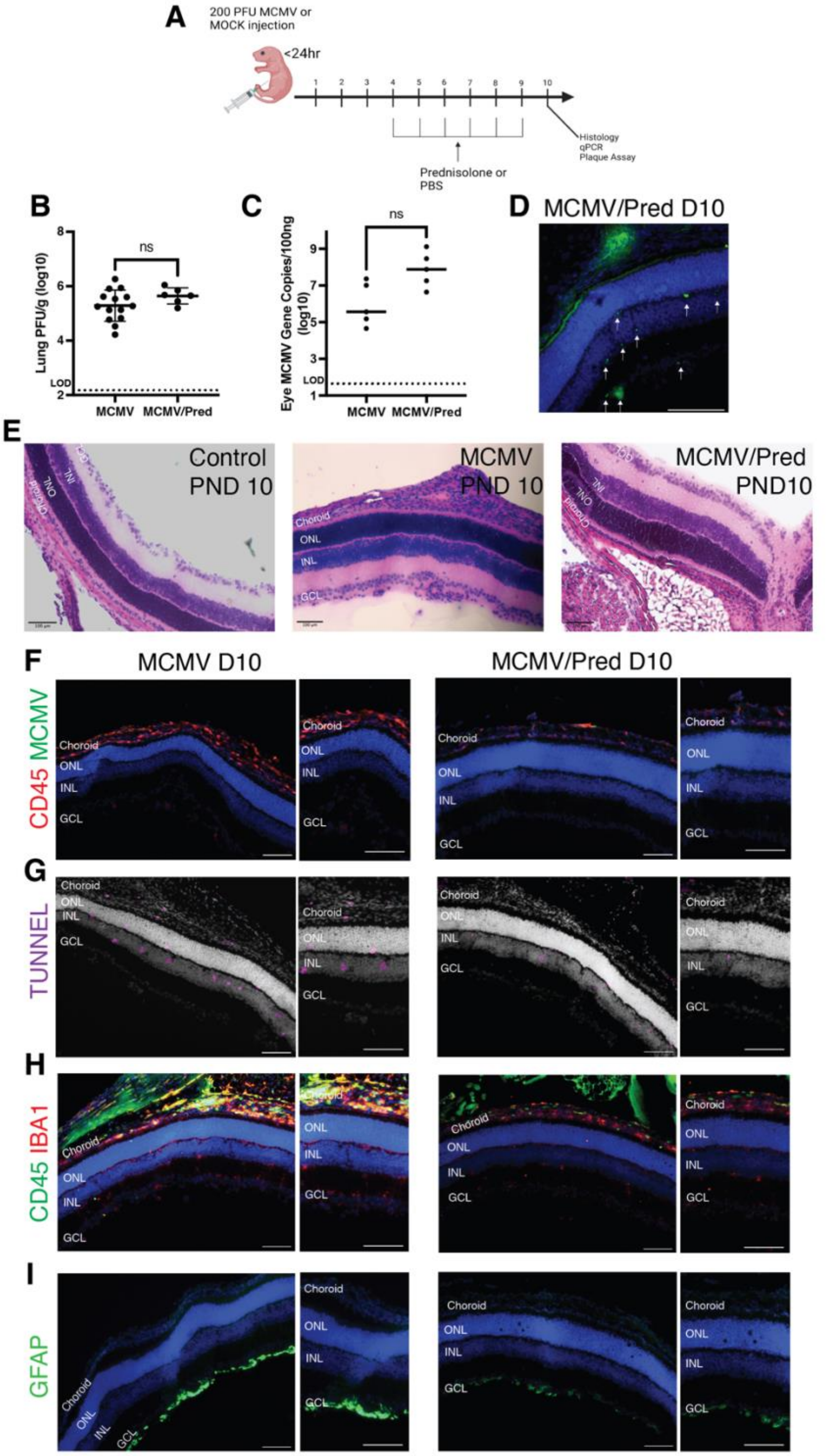

Fig S9: (A) Schematic of infection and prednisolone dose timing. **(B)** MCMV titer in lung tissue at PND10 of mice infected with MCMV and treated with prednisolone or PBS. Data was

measured by plaque assay and is shown as PFU/g lung tissue (LOD = 150 plaques/g). Analyzed by unpaired t-test; *$p < 0.05$ **$p < 0.01$ ***$p < 0.001$ ****$p < 0.0001$. **(C)** MCMV genome copies per 100 ng of DNA in ocular tissue at PND10 of mice infected with MCMV and treated with prednisolone or PBS (LOD = 44 genome copies/100ng). Statistics analyzed as in **(B)**. **(D)** Representative image of eye sections at PND10 from mice treated with prednisolone and stained for MCMV (pp89). White arrows denote $MCMV^+$ cells. **(E)** H&E staining of eyes at PND10 from control (uninfected) mice, MCMV-infected mice, and MCMV-infected treated with prednisolone. **(F-I)** Thin eye sections of eyes at PND10 from mice infected with MCMV and treated with prednisolone or PBS. A magnified area is shown to the right of each image. Sections were stained for: **(F)** CD45 and MCMV (pp89), **(G)** TUNEL+ cells, **(H)** CD45 and Iba1, or **(I)** GFAP. Scale bars indicate 100 µm. Data are representative of n=3-6 mice.

Fig. S10.

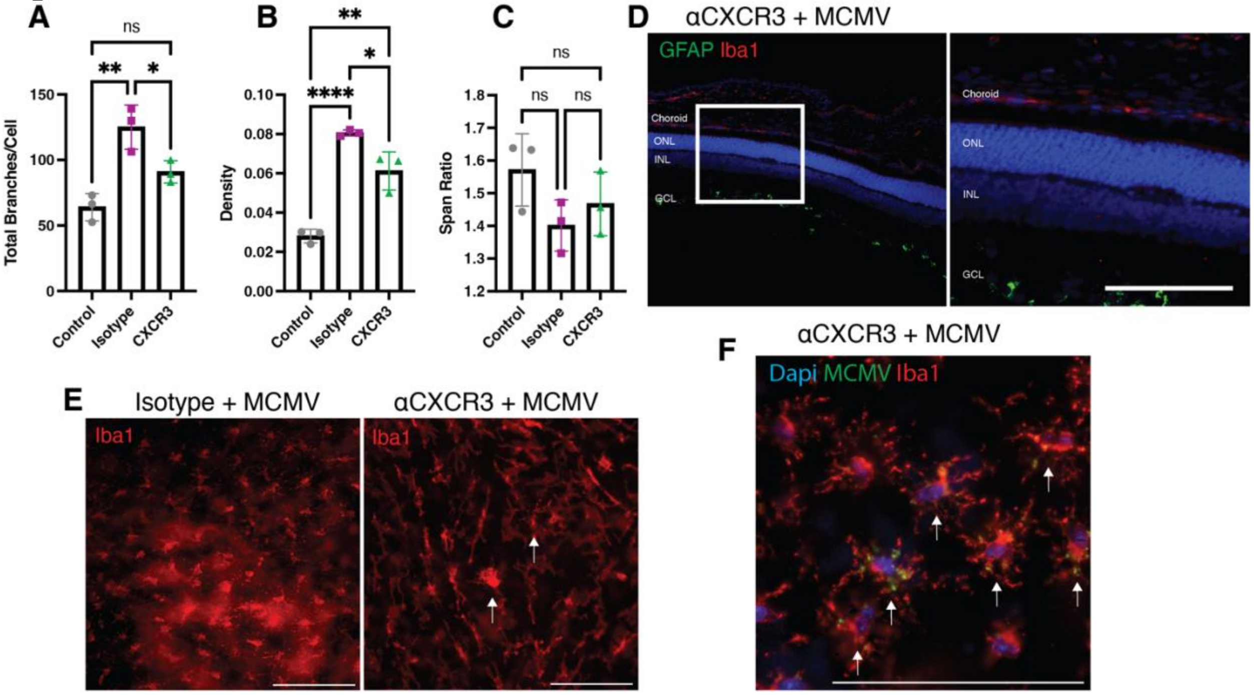

Fig S10: (A-C) Morphological analyses of Iba1+ microglia from control (uninfected) and MCMV-infected mice treated with isotype control, or αCXCR3 antibodies. Each data point shows an averaged value derived from n>15 cells from an individual mouse and was graphed from n=3 individual mice per group. Significance was analyzed by one-way ANOVA with multiple comparisons $*p < 0.05$ $**p < 0.01$ $***p < 0.001$ $****p < 0.0001$. Data show: **(A)** Total branches per cell, **(B)** Cell density, and **(C)** Span ratio. **(D)** Representative thin section of an eye at PND15 from an MCMV-infected mouse treated with αCXCR3 antibodies. Section was stained for GFAP+ and Iba1+ cells. Image on the right shows a magnified region (white box) to highlight the subretinal space. Data is representative of n=3 mice per group. **(E)** Flat mounts of RPE from MCMV infected mice treated with isotype control or αCXCR3 antibodies. Flat mounts were stained with Iba1 to show microglia in subretinal space. White arrows denote the only activated microglia found in the αCXCR3-treated mice (background Iba+ cells are tissue-resident macrophages in choroid). Data is representative of n=8 mice per group **(F)** Representative image of RPE flatmounts stained with DAPI, and for MCMV (pp89) and Iba1. The image shows microglia in the subretinal space stained positive for pp89 in cytoplasmic inclusions (white arrows). Data is representative of n=4. Scale bars throughout indicate 100µm.

Fig. S11.

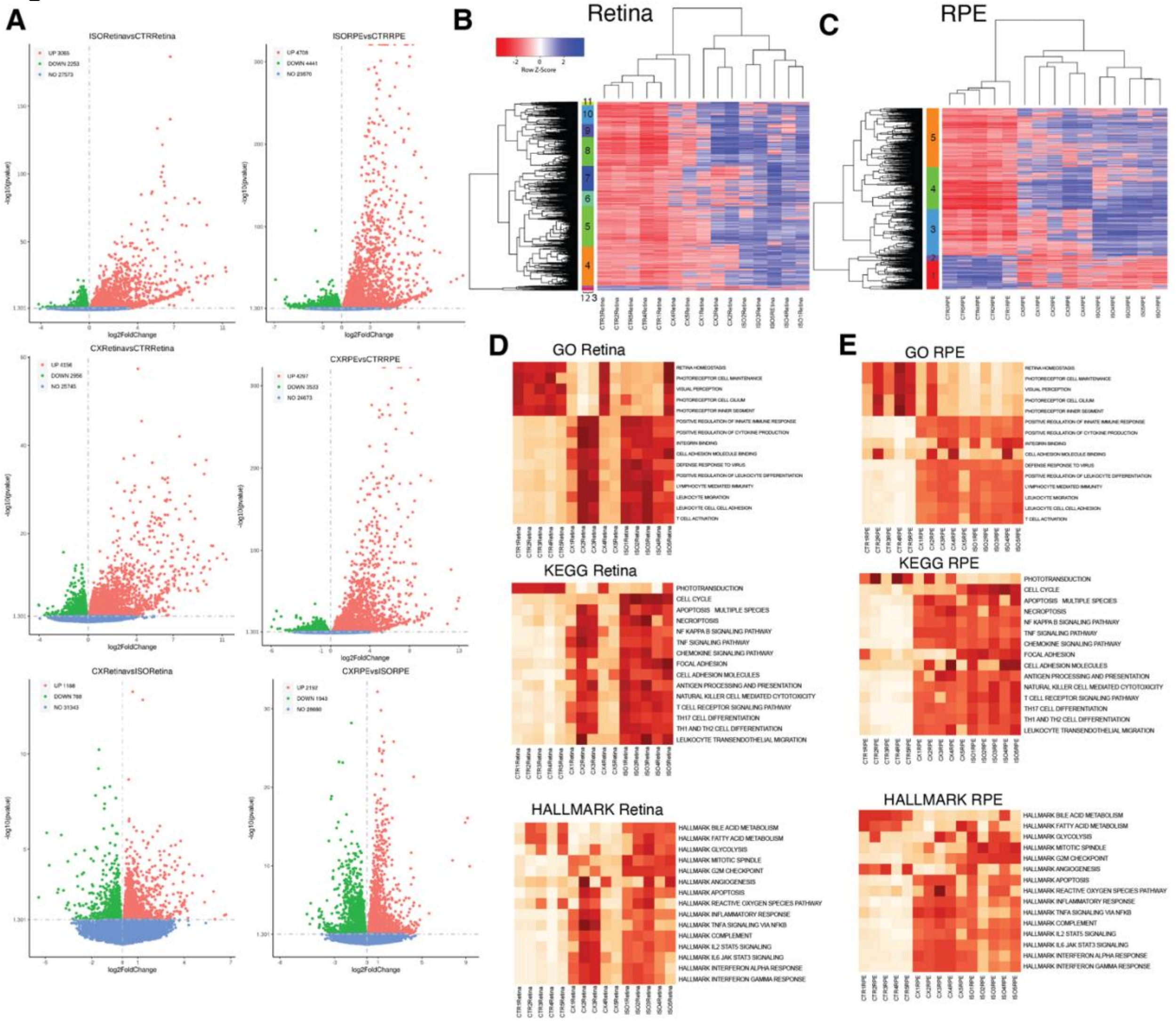

Fig S11: (A) Isolated retinas and RPE/choroids from uninfected mice (n=5, named CTR) or MCMV-infected mice treated with isotype control antibodies (n=5, named ISO) or αCXCR3 antibodies (n=5, named CX) were analyzed by RNA-Seq. Volcano plots show the differential gene expression between the indicated conditions. Data are plotted based on $\log_2$ fold change and log of the adjusted p-value. Red and green dots indicate significant positive and negative expression respectively (significance = >.05 p-value and > ±1 $\log_2$ fold change). **(B & C)** Heatmaps of GSVA enrichment scores against GO gene sets for each individual sample (CTR1-5, ISO1-5, CX1-5) from **(A)**. Individual pathway clusters determined by unsupervised clustering are labeled by number. Z-score is based on row values. **(B)** Heatmap of retina samples **(C)** Heatmap of RPE samples. **(D & E)** Pathways from GSEA analyses (GO, KEGG, and Hallmark gene sets) of MCMV+Isotype vs control uninfected and MCMV+ αCXCR3 vs control uninfected were selected for GSVA analysis. Heatmaps of GSVA pathway enrichment scores for each gene set were generated. **(D)** Heatmaps for GO, KEGG, and HALLMARK selected pathways for retina samples. **(E)** Heatmaps for GO, KEGG, and HALLMARK selected pathways for RPE samples.

Fig S12

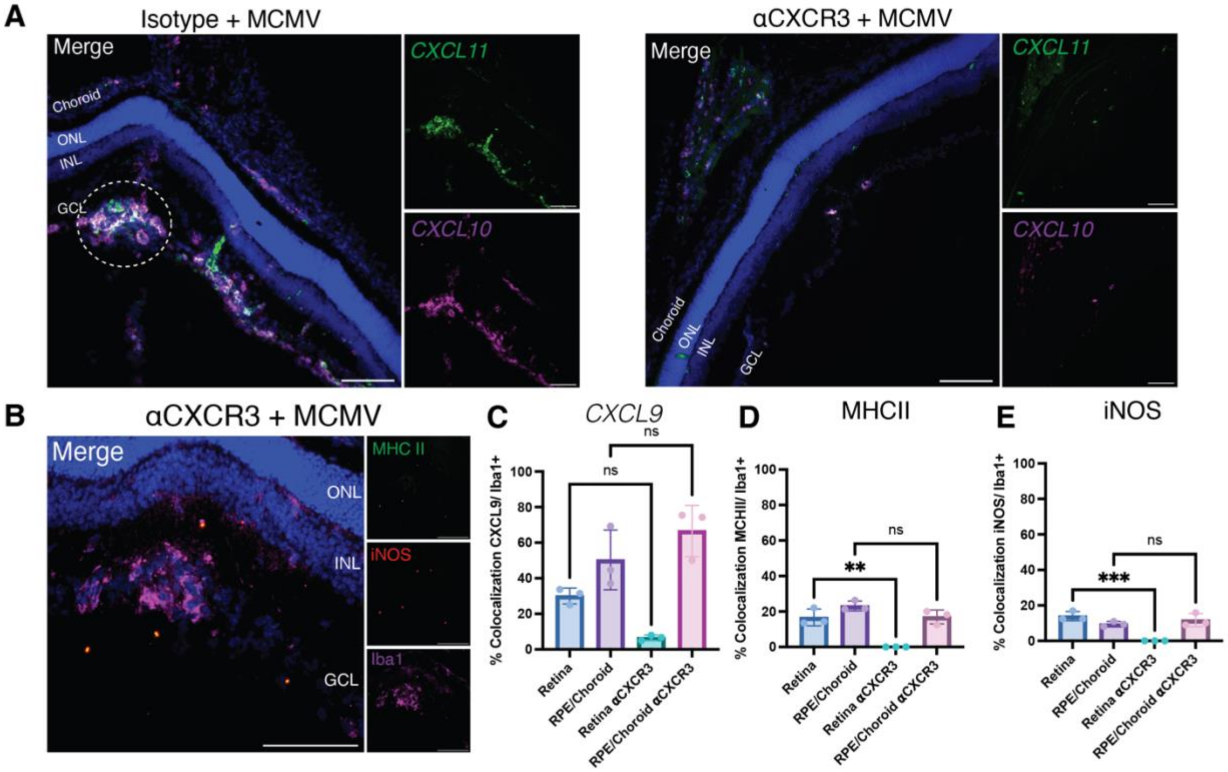

Fig S12: (A) In situ hybridization (RNA-scope) for *CXCL10* and *CXCL11* transcripts in eyes from MCMV infected mice treated with isotype control antibodies (left) or αCXCR3 antibodies (right). (**B)** Representative staining to identify activated microglia in an eye from an MCMV-infected mouse treated with αCXCR3. Section was stained for MHCII, iNOS and Iba1. (**C-E)** Colocalization of IFN-γ induced genes CXCL9, MHC-II and iNOS with with Iba1^{+} cells in the retina (microglia) and RPE/choroid (macrophages/monocytes). Data were collected from the entire retina or RPE/choroid in the section, including all cells staining positive for the indicated markers regardless of whether the cells were associated with a nodule or not. (**C)** % colocalization of all CXCL9 staining with all Iba1^{+} cells. (**D)** % colocalization of all MHC-II staining with all Iba1^{+} cells. (**E)** % colocalization of all iNOS staining with all Iba1^{+} cells. Data are representative of n=3 mice per group. Significance was assessed by one-way ANOVA with multiple comparisons *p < 0.05 **p <0.01 ***p < 0.001 ****p < 0.0001.

Supplemental tables

Table S1. Table of pathology observed in eyes of MCMV-infected mice over time. Data shown are the numbers of mice with the indicated pathology over the total numbers of mice observed.

Pathology	PND10	PND15	PND28
Eyes opened	0/18	4/23	9/9
Ruffled fur	14/14	22/23	6/6
Cellular infiltration of retina	4/16	16/16	9/9
Rosettes/Photoreceptor infoldings	7/11	6/8	4/6
Retinal layer disruption	12/16	14/14	9/9
Cellular clusters in GCL	14/16	16/16	8/9
Müller Glia Activation	1/10	11/11	6/6
Microglia Activation	8/8	16/16	7/7
RPE disruption	1/11	10/14	3/6
Inner BRB disruption (FITC-Dextran)	N/A	5/5	N/A
Outer BRB disruption (Occludin)	N/A	10/10	N/A

Table S2. Location of MCMV-stained cells with and without CXCR3 blockade.

Location	PND 15	αCXCR3 PND 15
**Choroid**	30.07%	21.78%
**RPE***	7.69%	9.27%
**IS/OS**	25.87%	26.97%
**Ciliary body**	8.04%	6.86%
**ONL*** **	1.05%	7.23%
**OPL**	1.40%	0.83%
**INL***	3.15%	11.68%
**IPL**	0.35%	0.09%
**GCL**	12.59%	10.10%
**Anterior**	9.79%	5.19%
**Total*<br>(avg. count)**	100%<br>(57.2)	100%<br>(119.89)

Table S2: MCMV infected all regions of the eye and the number of pp89^{+} cells was increased by CXCR3 blockade. Shown is the relative location of MCMV-stained (pp89^{+}) cells on PND15 with or without CXCR3 blockade. MCMV-stained cells in each anatomical region of the eye were counted. Shown are the percentages of pp89^{+} cells (nuclear staining and cytoplasmic inclusions) in each region as a proportion of all MCMV-stained cells across the eye. Significant increases in MCMV stained cells were observed in the RPE, ONL and INL leading to an overall increase in pp89^{+} cells in the eye (asterisks next to the location name). Numbers are an average of n=5 mice for PND15 and n=8 mice from αCXCR3 PND15. Data are from 4 thin sections per eye taken from ~100 um apart. Significant differences were identified by unpaired t-test; *p < 0.05 **p <0.01 ***p < 0.001 ****p < 0.0001.

Table S3. p-values of group comparisons generated from GSVA analyses on cell signature gene sets.

Cell Signatures	PND10 vs PND15	PND10 vs PND28	PND15 vs PND28	
HAY BONE MARROW CD8 T CELL	0.193314103	0.456030887	0.105232853	Lymphoid/T cell
HAY BONE MARROW NK CELLS	*0.097028128*	0.316838404	0.157883186	
TRAVAGLINI LUNG CD4 MEMORY EFFECTOR T CELL	0.22089749	0.305856169	0.222545752	
AIZARANI LIVER C34 MHC II POS B CELLS	0.354359572	**0.040865805**	*0.05163659*	
FAN EMBRYONIC CTX BRAIN NAIVE LIKE T CELL	**0.030781056**	0.543813527	**0.039896691**	
TRAVAGLINI LUNG CD8 MEMORY EFFECTOR T CELL	0.368606372	0.695465792	**0.026535156**	
TRAVAGLINI LUNG CD8 NAIVE T CELL	**0.012152194**	0.200930572	**0.031935836**	
GAUTAM EYE IRIS CILIARY BODY ACTIVATED T CELLS	0.213534331	0.377868855	*0.081489277*	
GAUTAM EYE CHOROID SCLERA ACTIVATED T CELLS	0.198264706	0.131434954	**0.000627054**	
DESCARTES FETAL LIVER MYELOID CELLS	0.601557507	**0.002451395**	**0.002038502**	Myeloid
DESCARTES MAIN FETAL MYELOID CELLS	0.990109652	**0.019803636**	**0.021925112**	
DESCARTES FETAL MUSCLE MYELOID CELLS	0.183791679	**0.013683718**	**0.010879411**	
TRAVAGLINI LUNG NONCLASSICAL MONOCYTE CELL	0.500359416	**0.010388236**	**0.009110538**	
TRAVAGLINI LUNG CLASSICAL MONOCYTE CELL	0.219665316	**0.005344525**	**0.005925387**	
DESCARTES FETAL LUNG MYELOID CELLS	0.461782107	**0.032537068**	**0.03357704**	
HE LIM SUN FETAL LUNG C2 CXCL9 POS MACROPHAGE CELL	*0.094892914*	**0.019618177**	**0.007222717**	
DESCARTES MAIN FETAL MICROGLIA	**0.003882105**	**0.005895911**	**0.001153963**	Microglia
DESCARTES FETAL EYE MICROGLIA	0.232219202	**0.033149216**	**0.016037194**	
ZHONG PFC MAJOR TYPES MICROGLIA	**0.01969385**	0.104011739	**0.013614185**	
FAN EMBRYONIC CTX BIG GROUPS MICROGLIA	**0.009441253**	*0.058404405*	**0.011424061**	
HU FETAL RETINA MICROGLIA	**0.01346321**	**0.072850051**	**0.008039614**	
DESCARTES FETAL CEREBRUM MICROGLIA	**0.005737866**	**0.008704153**	**0.002831602**	
DESCARTES FETAL CEREBELLUM MICROGLIA	**0.015498433**	**0.039920197**	**0.009889966**	

Table S4. Table of Antibodies used.

Antigen	Species	Supplier	Product no.	Dilution
Iba1	Rabbit	Wako	019-19741	1:1000
CD45	Rat	Biolegend	103123	1:200
CD8	Rat	Biolegend	100708	1:200
GFAP	Mouse	Invitrogen	53-9892-82	1:500
Occludin	Mouse	Invitrogen	331588	1:200
MHCII(I-Ad)	Mouse	BioLegend	115008	1:200
iNOS	Rat	Invitrogen	14-5920-82	1:500
pp89 (MCMV IE1 gene)	Mouse	Snyder lab	Clone 6/58/1	1:200
Anti-Rabbit IgG	Donkey	BioLegend	406421	1:200
Anti-Rat IgG2a	mouse	Biolegend	407509	1:200
Anti-CXCR3-173	Armenian Hamster	BioXcell	BE0249	N/A
IgG isotype control (PIP)	Armenian Hamster	BioXcell	BE0260	N/A

Supplemental Movies

Movie S1. 3D projection of retina flat mount from an MCMV infected mouse on PND15, stained for GFAP (Top right), Iba1 (Bottom left), and CD8 (bottom right) (Merge Top left).

Supplemental Data

Data file S1. List of genes in each cluster from co-expression cluster analysis.
